# Comparing the Use and Usefulness of Four IoT Security Labels

**DOI:** 10.1145/3613904.3642951

**Published:** 2024-05-11

**Authors:** Peter J. Caven, Zitao Zhang, Jacob Abbott, Xinyao Ma, L. Jean Camp

**Affiliations:** Indiana University Bloomington, Bloomington, Indiana, USA

**Keywords:** labels, security, privacy, trust, interaction, IoT, icons

## Abstract

There are currently multiple proposed security label designs for consumer products, with each prioritizing different security and privacy factors. These differences risk making product comparisons more confusing than informative. Standardized labels could potentially resolve this by informing consumers of a product’s security features at the point of purchase. But which standard? This survey, of 500 participants, studied four label designs and measured comprehension, response time, acceptability, and cognitive load. We gauged understanding of participant perception and preferences using three smart devices: light bulbs, cameras, and thermostats. We identified preferences and behaviors before, during, and after label use for product selection. At first, participants believed more information-dense labels would better support their purchasing behavior; however, after they evaluated and compared products, participants gravitated towards less cognitively demanding designs. We identified how participants utilized and prioritized label elements to provide recommendations for US label design efforts.

## 1 INTRODUCTION

Security labels enable consumers to make purchases informed by security quality, and the United States has taken recent steps to increase this transparency. In July of 2023, the Federal Communications Commission (FCC) introduced the U.S. Cyber Trust Mark as part of a voluntary security labeling effort for smart devices [22, 61]. This program is the culmination of efforts derived from President Biden’s 2021 Executive Order on Improving the Nation’s Cybersecurity (EO 14028) [31]. Its importance was again reified in the 2023 National Cybersecurity Strategy [62]. These efforts are an essential first step in protecting the US’ security ecosystem, as human decisions are a critical component of computer security [40, 55]. Prior to this program, Underwriters Laboratory developed a five-level security ranking, indicated by shield icons ranging from bronze to diamond [66]. However, these are not the only labeling efforts. Researchers have proposed nutrition-style labels [36], icons [69], eyes [57], and various other label designs [15, 18, 23, 59]. These disparate efforts emphasize that unification is essential to enhancing consumer trust and the legitimacy of security claims. Standardized security labels will enable consumers to make informed choices and increase the likelihood of manufacturer compliance.

As the U.S. Cyber Trust Mark is primarily a US initiative, this study uses a representative sample of US demographics. We report on changes to participants’ perceptions of usability and acceptability using four different label designs: binary, graded, descriptive, and multi-layer. These designs were selected as they were proposed for consideration by the Consumer Technology Association’s label working group as possible solutions to resolve information asymmetry. Additionally, these designs are all different from one another, representing distinct types of consumer-oriented labels, which we discuss in detail in Section 3. To evaluate perceptions of usability, we conducted a survey across three smart devices: light bulbs, cameras, and thermostats. The goal of this research is primarily to gauge the effectiveness of proposed IoT security labels in informing purchasing behaviors. Considering purchasing decisions are often not unidimensional (i.e., they include other factors such as price, quality, or features), we also examine the mental workload and its associated influence on label preference. We evaluated participant accuracy, response time, cognitive load, and perception of the information provided by each design. Quick Response (QR) codes have been proposed in conjunction with binary or graded labels to act as a redirect to additional security information [10, 18, 51]; however, prior studies have shown QR codes to not be very effective due to low practical utilization [6, 33]. While inherently limited given the modality of the study, we also attempt to evaluate and quantify the use of QR codes.

The results from this study are relevant to US security and privacy researchers and policymakers, especially as online risks continue to grow in complexity and frequency [2, 32, 45, 71]. Risk communication is a challenge. The constant evolution of technology means new risks constantly emerge, making it difficult for US consumers to remain vigilant. As threats continue to evolve, tailoring information to varying levels of expertise and awareness poses a significant challenge. Bridging this gap between technical safeguards and user comprehension is the goal of any security label. However, to what point is a label useful and actionable without becoming overwhelming or counterproductive? Finding this balance is crucial. A security label should empower consumers. It needs to be both informative and user-friendly, ensuring that consumers can trust the security quality without feeling overwhelmed. Risk communication is a challenge that stems from the dynamic nature of the technology ecosystem, which necessitates adaptive, accessible, and psychologically informed communication strategies to effectively convey risks to diverse consumers.

As we will see from our experiment, adding more information does not necessarily increase the effectiveness of a security label’s ability to inform security decisions. In some cases, it increased the cognitive workload and time to make decisions, while not significantly increasing security accuracy. As the US government attempts to keep pace with the rapidly evolving technology landscape, labeling efforts should be built to meet the future needs of consumers. By focusing on the underlying perceptions, motivations, and behaviors that underpin individuals’ responses we gain an emergent understanding that can drive security and privacy discourse. This can be leveraged to develop more targeted and impactful risk communication strategies. Rather than inundating consumers with more information, security labels should strive for clarity and relevance. Concisely conveying information allows labels to become more than just seldom-used artifacts, it transcends security labels to be impactful points of difference that enable US consumers to make more secure choices.

## 2 RELATED WORK

Labels are a communication mechanism, so their effectiveness is contingent on the recipient. But for any label to be effective, it is critical to provide a design that supports a basis for comparison. The difficulty of designing a universal label for all stakeholders is obvious. An appropriate label is a function of the information needed by the user, the level of technical acumen, and the role of the user in the ecosystem. While security, not privacy, was the motivating factor of EO 14028 [31], lessons learned from privacy research are still salient when considering the security labeling initiative. Apropos of the newer security label efforts, privacy has long been the primary driver for computing usability and interaction research. If privacy is the goal, then security is the enabler. Therefore, any development of security labeling efforts should consider these similar but disparate concepts. In this regard, we will review previous research on privacy policies and Apple’s Privacy Nutrition Label.

Prior to privacy labeling efforts, privacy policies were used to inform consumers about data and security risks. However, despite their ubiquity, these privacy policies were found to be generally ineffective. Studies have consistently identified both length and complexity as reasons for this usability failure [34, 42, 53, 56, 63, 67]. To remedy the readability and usability challenges in privacy policies, there have been efforts to standardize a single privacy framework and provide relevant, timely, and actionable information at the point of purchase [7, 56]. Motivating the argument for labels, researchers have claimed that the notice and choice model of privacy protection is inadequate; instead, leveraging transparency and privacy by design to promote trust in products is needed [14, 60]. The same ideas are applicable to security. Labels would ideally make security straightforward for even the most non-technical consumer, leveraging intuitive graphical design [10, 14, 44].

**Figure 1:**
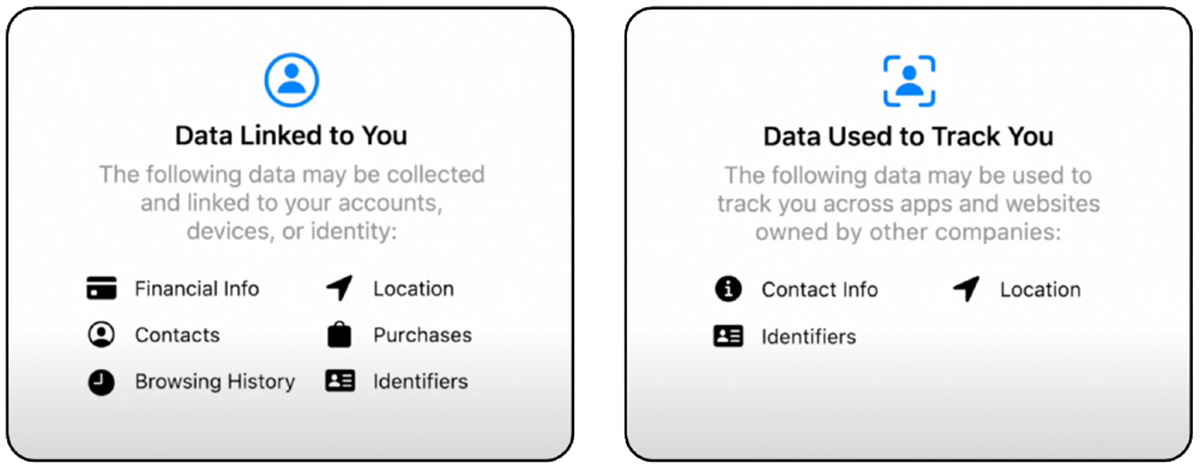
Screenshots of Apple’s Privacy Nutrition Label (PNL). While there is valuable information, it may be vague, too much, or too complex to truly inform regular users.

These early privacy efforts paved the way for Apple’s Privacy Nutrition Label (PNL), which became a requirement in December 2020 for all apps available on Apple’s App Store [3, 38]. Apple’s PNL, shown in Figure 1, is an online label that provides users with information about how an app collects and uses personal data. This is part of the company’s broader push for increased privacy and data protection [4]. The label’s goal is to empower consumers by providing them with more understandable information about an app’s data practices, allowing them to make privacy-conscious decisions prior to downloading. This initiative aligns with some of the best practices from previously mentioned privacy policy research, which increases consumer awareness about data privacy: simplifying language, providing a simple dashboard of relevant information, information at the time of download, standardizing the information for all hosted apps, and encouraging developers to be more transparent with their data handling practices. This is reflected by the increased demand for apps using labels that touted non-identifiable and non-personal information, as the app was perceived as being more trusted [26].

However, as applications continue to become more complex and intertwined, PNLs are not without their challenges. Developers need to be aware of how their app manages data (e.g., collect, use, and share), be able to interpret PNL data requirements, accurately reflect the app’s data practices, and make it accessible to users [24, 38]. Apple’s PNL is an important step toward protecting user privacy, but more work is needed to make it more effective. Some users found the labels to be helpful; though most found them to still be too long, complex, or confusing; while others found them to be outright misleading, due to developers not accurately reporting their data practices [37, 72]. Apple’s Privacy Nutrition Label has had a significant influence on how consumers perceive and interact with privacy sensitive information. Security label designs should leverage these real-world applications and research to ensure usability. Next, we will review four security label designs, and how they attempt to resolve similar issues rooted in the security domain.

## 3 SECURITY LABEL DESIGNS

Prior researchers have recognized four primary categories of consumer-oriented labels: binary, graded, descriptive, and multi-layer labels [8, 10, 12, 18, 25, 27, 35]. We map these categories to the four tested label designs:

- United States Cyber Trust Mark based on binary labels [61]
- Underwriters Laboratories (UL) IoT Security Rating based on graded labels [66]
- Carnegie Mellon University (CMU) IoT Security and Privacy Label based on descriptive labels [17]
- We propose a Five-Lock Label based on multi-layered labels

While the CMU IoT Security and Privacy Label also incorporates aspects of a multi-layered label, we wanted to evaluate the efficacy of a more obvious design. To do this we developed a graded multi-layered label, based on the gaps within current privacy labeling efforts and previous security labeling/icon design research [54, 57]. This internal graded system is similar to five-star ratings that proliferate the web (e.g., Amazon, eBay, Uber, and AirBnB), and its development is discussed in more detail in the next section. In our experiment, we have working QR codes for both the CMU and Five-Lock multi-layered labels.

It is important to keep in mind that the depth of information is contingent on the type of label employed. Each label has distinct strengths and weaknesses, and we now will briefly examine these and their interactions in consumer decision-making.

### 3.1 Binary Label

**Figure 2:**
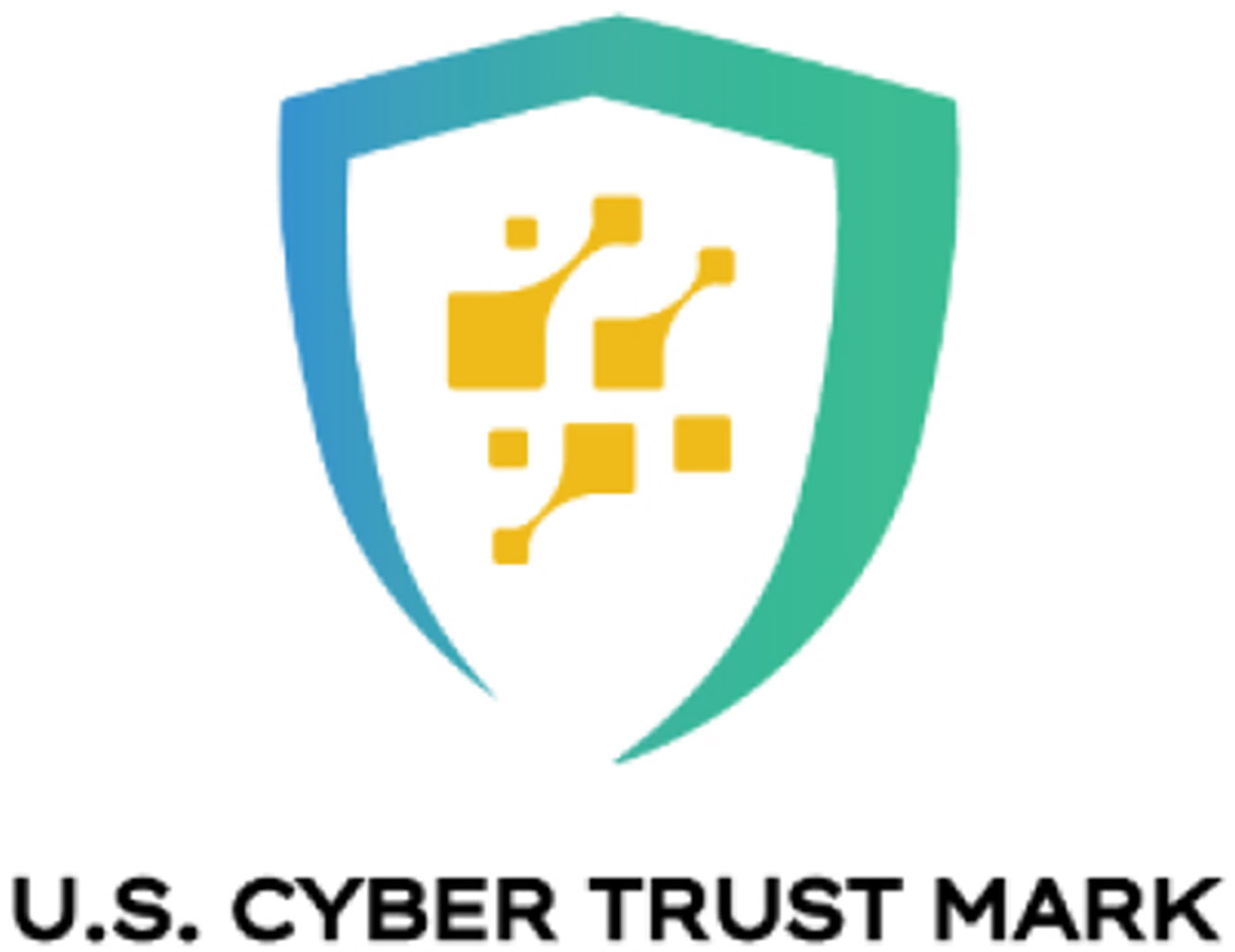
The U.S. Cyber Trust Mark is a binary label that ensures the baseline compliance of a security standard [22]

The *U.S. Cyber Trust Mark* was announced by the FCC in July 2023 [22]. This binary label is proposed as a voluntary program designed to help consumers make more informed decisions about smart devices [61]. This effort was an extension of the National Institute of Standards and Technology (NIST) development of baseline standards in 2021 [48] and 2022 [49]. The U.S. Cyber Trust Mark label uses a standardized shield logo, which would be displayed on qualifying products, acting as a certifying seal. This is depicted in Figure 2. Future versions may use a multi-layer model, linking to more detailed information. We refer to the U.S. Cyber Trust Mark as the **Binary label** in the remainder of this work. The Binary label does not present individual risk factors, as it only indicates that labeled products pose less risk than unlabeled products.

The simplest approach to mitigate information asymmetry is using a Binary label. If a product meets specific security criteria, it earns a label, acting as a seal of approval for security quality [25]. This is by far the simplest way to ensure the security of an ecosystem and can be assessed through the lens of usability heuristics. Binary labels adhere to the usability heuristic of visibility of system status [52]. This means that it effectively conveys information without burdening the user with excessive cognitive load. However, this simplicity prevents it from conveying any nuanced security information, as all products appear secure despite only meeting the minimum thresholds [35].

### 3.2 Graded-Shield Label

**Figure 3:**
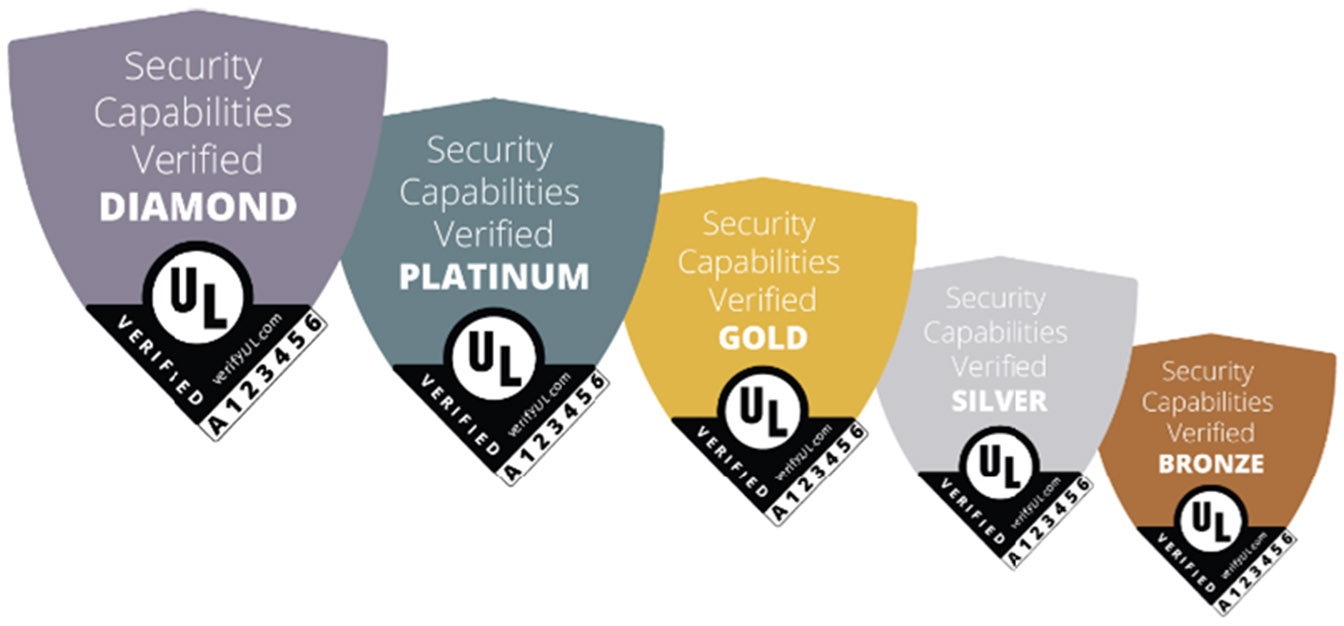
The UL IoT Security Rating is a graded label using colored shields to represent the security quality of a product [64]

Underwriters Laboratories’ *IoT Security Rating* program was first introduced in 2019, based on its IoT Security Top 20 Design Principles [64, 65]. These graded labels assure the security claims of a product using an ascending five-level scale labeling guide and are represented in Figure 3: Bronze (Essential), Silver (Enhanced), Gold (Advanced), Platinum (Extensive), and Diamond (Comprehensive) [66]. Each labeling tier is validated by UL and represents manufacturer compliance with distinct security features used in the device. We refer to the IoT Security Rating as the **Graded-Shield label** in the remainder of this work. The Graded-Shield label also does not present individual risk factors; however, unlike the Binary label, it presents different levels of potential risk, indicating higher security and lower risk.

Graded-Shield labels use a rating scale similar to a 5-star scale to vouch for the security quality of the product. However, unlike a 5-star scale, this instantiation is not internally graded. Instead, consumers have to either have an understanding of the scale or visually see other labeled products to frame a product’s rankings. When all information is available, research has shown that higher-scored devices are more likely to be purchased [35]. However, unlabeled devices were more likely to be purchased when compared to low-scored devices, which is concerning for a voluntary labeling program as it would incentivize removing labels for poorly scored products [19, 25, 35].

### 3.3 Nutrition-Style Label

**Figure 4:**
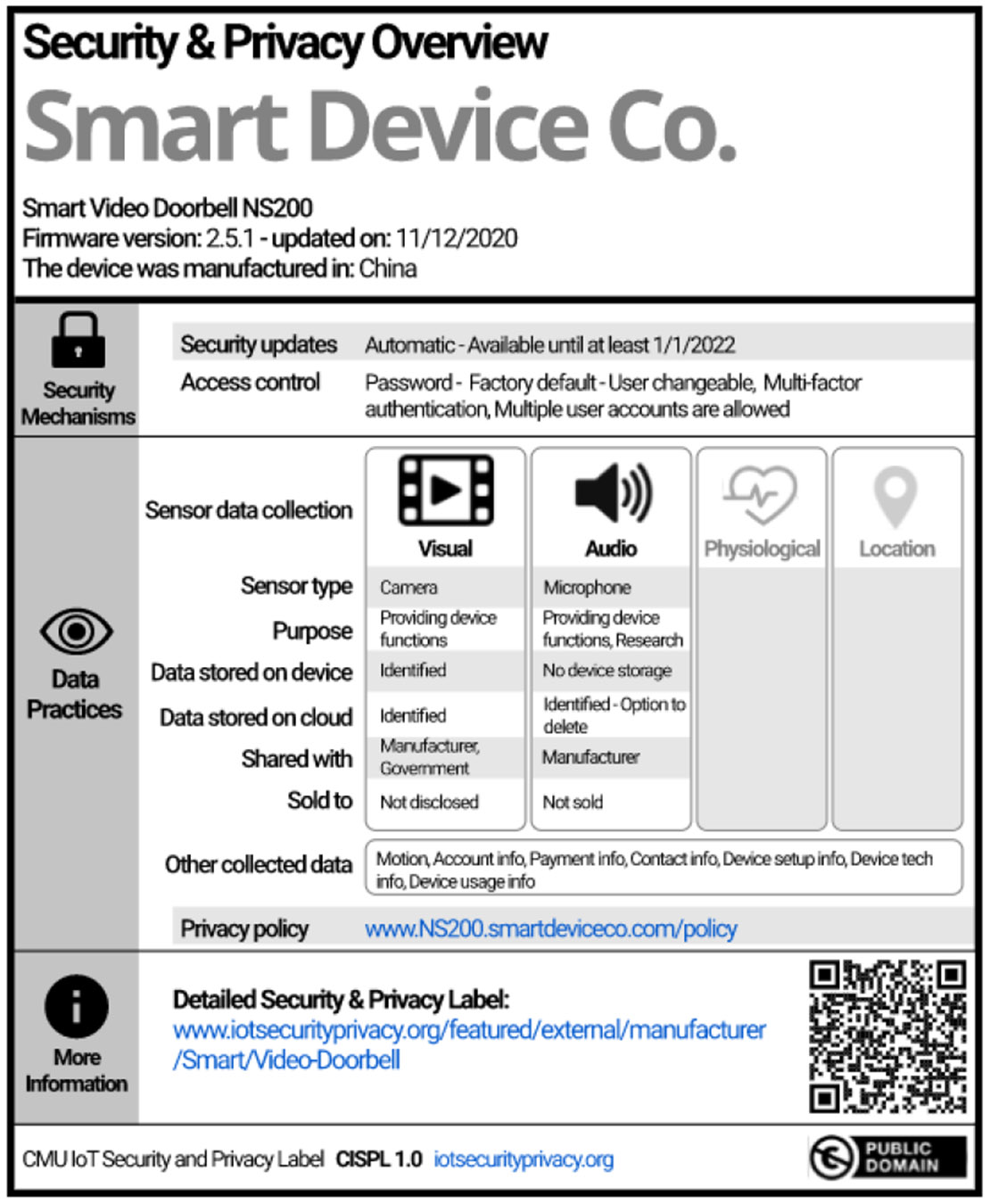
The CMU IoT Security and Privacy Label is a descriptive multi-layer label similar to nutrition labels [17]

In 2020, Carnegie Mellon University (CMU) introduced a standard way for manufacturers to communicate the security and privacy practices of their IoT devices by using its *CMU IoT Security and Privacy Label (CISPL)* [17, 18]. This label is represented in Figure 4. The descriptive label leverages a multi-layer model. The primary layer includes a straightforward listing of the product’s security, and a more detailed layer accessed via URL or QR code. The primary layer is shown in Figure 4, and both the primary layer and secondary layer are shown later in this paper, in Figure 7. We refer to the CISPL as the **Nutrition-Style label** in the remainder of this work. The Nutrition-Style label presents each individual risk factor to users; however, it may be difficult to use without a comprehensive understanding of technical jargon.

Many governments have explored using a similar type of label to list a product’s security and privacy information due to the ability to directly display pertinent information [25]. However, doing so may make Nutrition-Style labels long or complex, while still not fully indicating the level at which the product has met or exceeded a standard. These factors make product comparisons difficult. Still, these labels provide much more information and can be evaluated in terms of recognition rather than recall [52]. With the essential information directly on the label, this aligns with the principle that users should not have to commit information to memory, but they should be able to recognize it readily. However, the label appears to fall short in adhering to the heuristic of aesthetic and minimalist design [52]. This deficit in design aesthetics may impede its overall usability and user-friendliness. Nonetheless, researchers found some consumers, especially more technical ones, preferred a design that offered more information [33, 46].

### 3.4 Five-Lock Label

**Figure 5:**
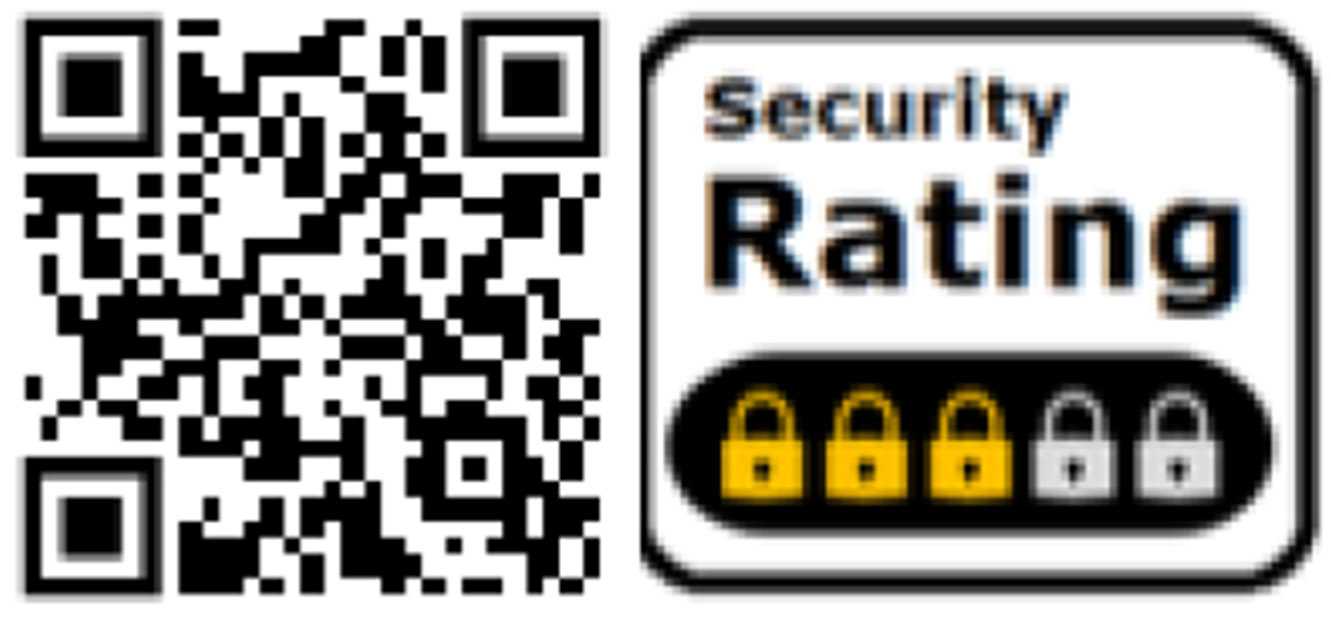
The Five-Lock label is an internally graded multi-layer using a QR code

We also test a proposed *graded multi-layer label*. Instead of using the ubiquitous five-stars common across most rating designs, we use a five-lock design, shown in Figure 5. Locks are strongly associated with security; users often misinterpret the security guarantees of a lock icon in a web page’s address bar to extend to all site security metrics [68]. Leveraging this intuitive reliance on lock icons as a point of difference can easily communicate levels of security for purchasing decisions. Studies leveraging online platforms have found that simple five-lock indicators would positively influence secure purchase decisions [54, 57]. Like the Nutrition-Style label mentioned above, this label is also multi-layer, as it similarly includes a QR code pointing to a second layer that contains additional security details. However, the Five-Lock label has a clearer design language in that the QR code is the same size as the representative label. This label’s underlying security context is based on information from meta-studies of security practices [11, 13, 16-18, 43] and federal guidelines [47, 49, 50, 58]. We refer to this as the Five-Lock label. The Five-Lock label can present different levels of risks to different users; it shows comparable information to non-technical users while pointing more technical users toward pages with more detailed security information.

The Five-Lock label minimizes the negatives of the previous label designs. By leveraging multi-layers, it can target different population segments by displaying varying amounts of information at different levels of the label. Researchers who have combined simple indicators with more technical information found it to be the most effective at encouraging risk-aware consumer decisions [28, 57]. Simple indicators on the first layer could be targeted toward non-technical consumers, while the second layer has more detailed information such as security specifications or device implications that target consumers with a higher level of understanding of terminology regarding cybersecurity. Multi-level labels have the potential to address information asymmetry and bounded rationality by providing access to information, while at the same time facilitating product comparisons [10, 17, 18].

## 4 ADDITIONAL INTERACTIONS

Consumers interact with products through various channels and labels should be similarly designed for both online and offline use. Research has indicated that a significant portion of smart device consumers make purchases through online channels [19, 20]. However, space for labels on physical products are more constrained due to product packaging. Multi-layer labels emerge as a strategic solution to provide depth of information. We briefly examine how labels can be leveraged by online marketplaces, as well as using QR codes to achieve information depth. These concepts will be carried forward into our study design in Section 5.2.

### 4.1 Marketplace Interaction

Online marketplaces in the US, such as Amazon, provide consumers with a variety of products offered by a diverse set of sellers [73]. Utilizing a digital interface, there is no limit to the amount of information that a seller can provide about a product. But with no standardized way of contextualizing or communicating security, how do consumers evaluate it (if at all)? Current security information failures stem from this lack of comparability, complexity, jargon, and user-friendly presentation [36]. The non-standardized security disclosures across online marketplaces make it challenging for a consumer to evaluate risks effectively. The use of any standardized security communication (i.e., any of the label designs discussed above) is a way online marketplaces can consistently communicate security, both internally and externally.

**Figure 6:**
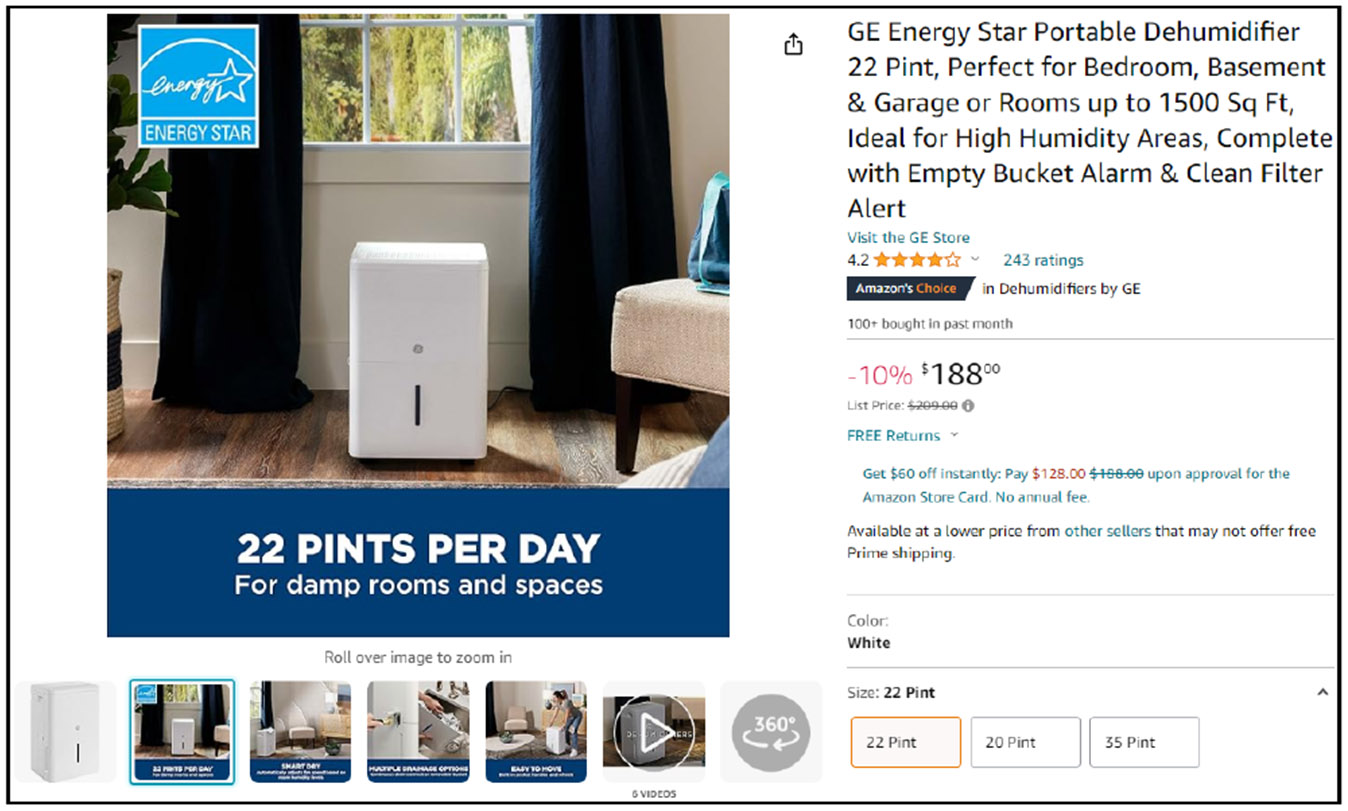
A dehumidifier listing from Amazon that uses “Energy Star” in the product title as well as the Energy Star label on product imaging [1].

Security labeling efforts can draw on similarities to the Energy Star labeling program. Energy Star was established by the Environmental Protection Agency (EPA) in 1992 as a voluntary labeling program to help physical businesses communicate energy efficiency information to consumers [21]. The Energy Star label has successfully set a precedent for communicating intricate information. As a significant point of difference, consumers leverage this label to find products that match their purchasing preferences. This ubiquity has helped it bridge the gap between physical and online markets, as shown by its use in a digital marketplace in Figure 6. Aligning to a similar strategy of providing both online and offline consistency, security labels used online should be the same as their physical store counterparts. Just as the Energy Star label aids consumers in making environmentally conscious choices in both online and offline markets, security labels can contribute to decision-making regarding the safety and privacy of products. While online markets might be the way most consumers purchase smart devices [19, 20], physical markets will drive the overall design. Considerations need to be made for the constraints of limited space on product packaging.

### 4.2 QR Codes Interaction

In this study, two label designs leveraged QR codes to provide more security information about a product (i.e., the Nutrition-Style label and the Five-Lock label). Multi-layer labels can use QR codes to point to more technical information and have been found to be more effective at encouraging risk-aware procurement decisions [28, 57]. However, prior research has shown a low utilization of QR codes [6, 33].

Still, secondary layers may be beneficial to more technical consumers who are making more complex or higher-level integration decisions. As these are consumers who desire more information, superficial or baseline labels may not adequately satiate their information need [33, 46]. However, a low engagement rate suggests that, despite the potential benefits, there are significant design barriers or disincentives preventing utilization. This could be attributed to factors such as a lack of awareness, perceived complexity, or an unwillingness to engage with QR codes.

**Figure 7:**
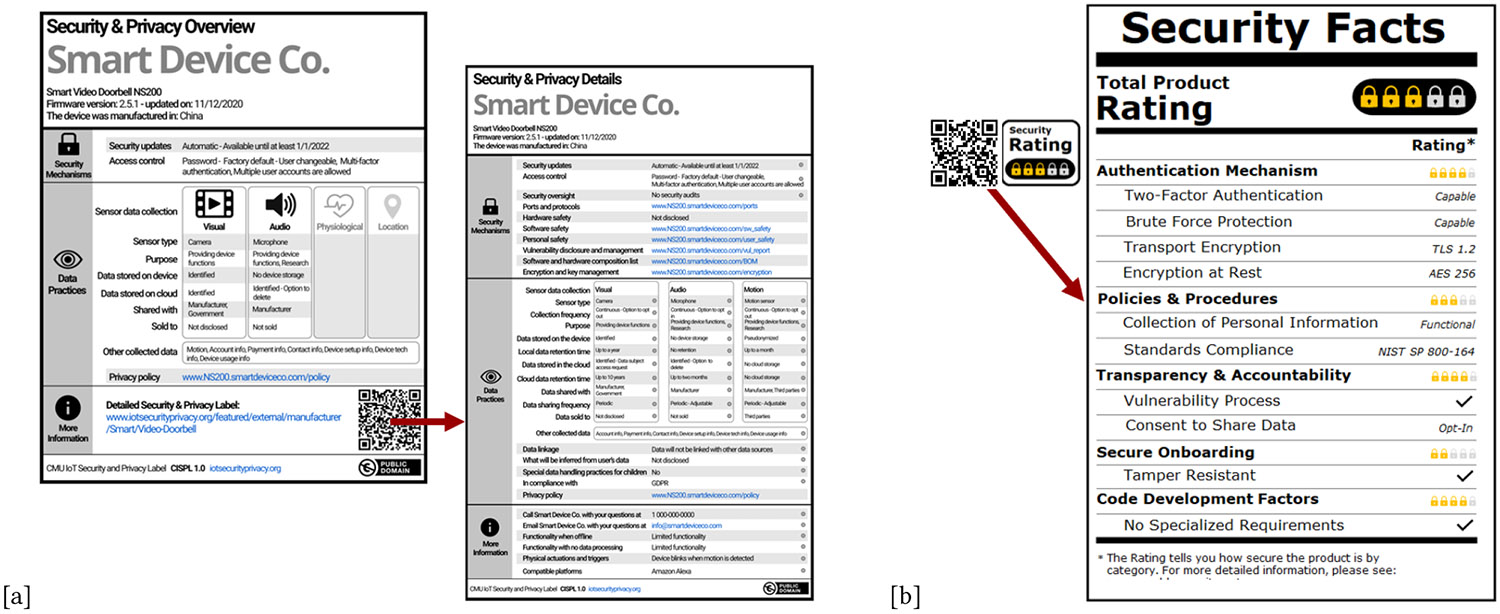
Primary and secondary layers for both multi-labeling designs we tested within this experiment: (a) the Nutrition-Style label is a descriptive multi-layer label [17], and (b) the Five-Lock label is a graded multi-layer label.

For our study, we test QR code usage by hosting working secondary layers and tracking their views. An example of both layers for Nutrition-Style and Five-Lock label designs are shown in Figure 7. Secondary pages are linked to the primary label through QR codes on a separate webpage, and view counts are tracked. While we acknowledge the inherent constraints of online interactions replicating physical interaction, this method can still provide a valuable avenue for assessing user interactions with QR codes. Simply providing hypothetical usage questions may result in the over-representation of optimistic responses [9]. Using QR codes in this study allowed us to capture quantifiable data to supplement hypothetical QR code usage questions. Additionally, it forces participants to reflect on their actions and usage, and this tacit feedback may more accurately represent true sentiments about QR code preferences.

## 5 METHOD

In this section, we review the experiment design and demographic information of our survey participants. Our findings can shape and inform discussions surrounding the current development of the US’ labeling framework [61]. The implications of this research extend beyond academia; the results can foster a deeper understanding among policymakers, industry stakeholders, and consumers. This could potentially lead to practical improvements in the way labels are designed and communicated. As technology continues to evolve, this research provides a timely contribution to the ongoing efforts to balance innovation with user security and privacy.

### 5.1 Research Questions

The purpose of this research is to analyze and identify which labels are salient and acceptable. In order to gauge the effectiveness of a label’s ability to inform consumers, we sought to address the following questions:

**RQ1:** Which type of label is more effective in conveying security information and influencing purchase behaviors?

To measure this we asked participants to select the most secure device when presented with different labels.

**RQ2:** Which label design is less mentally taxing to use?

To measure the participant’s task burden we used the NASA-TLX measurement. We also measure the time for each participant’s decisions.

**RQ3:** Will consumers reliably use QR codes to inform their decision-making?

For the labels with QR codes, we linked these codes to pages that captured their use. We also asked them about their QR code habits.

**RQ4:** What labels do participants prefer, and does use change that preference?

We asked participants at the start of our survey which design they felt ensured security. At the end of the study, we ask this again.

We used standard tools and techniques (e.g., surveys, click counts, page views, page submission times, qualitative coding, ANOVA, etc.) to obtain measures of participants’ opinions, behaviors, and accuracy when interacting with the labels. This research consisted of questions relating to label preferences, security knowledge, Task Load Index (TLX), product selection influences, and demographics. All experimental protocols were approved by Indiana University’s Institutional Review Board (IRB) before recruitment. The survey was built using Qualtrics and hosted on Prolific. Prior to release, the survey was piloted within our research lab, where we established time to completion and sequencing of questions.

### 5.2 Study Design

Our study was conducted online and asynchronously. Participants were not given any extra information or instructions about the labels, aiming to replicate real-world usage and better assess label usefulness. While inherently limited given the modality of the study, we also measured QR code usage by hosting a secondary label and tracking page visits. In total, there were eight distinct phases to this study, depicted in Figure 9, and, on average, participants were expected to complete the entire study in under 20 minutes. An excerpt of the study protocol can be found in Appendix B.

**Figure 8:**
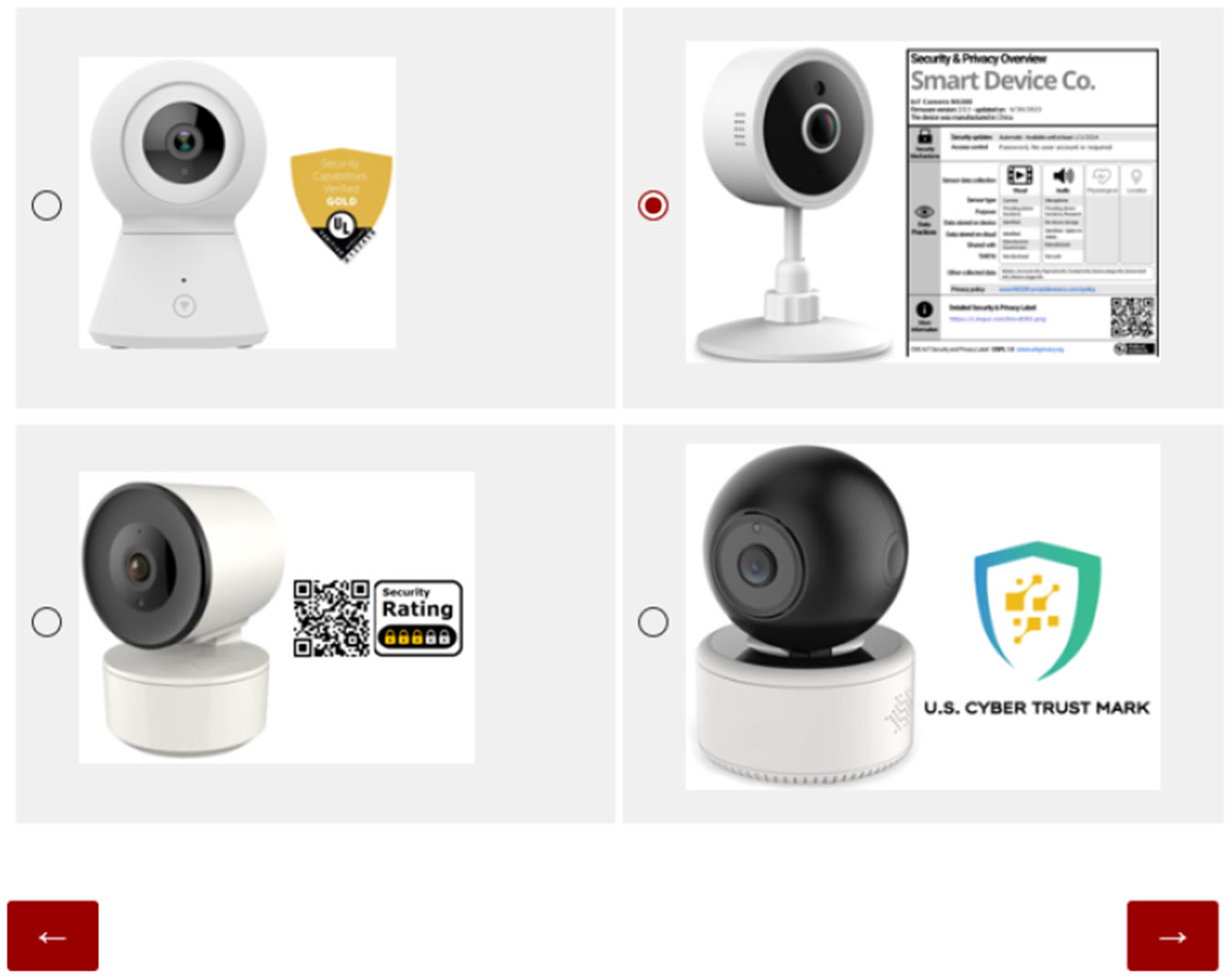
Participants were presented with four camera options in Phase 2 and asked to select the one offering the greatest cybersecurity.

**Figure 9:**
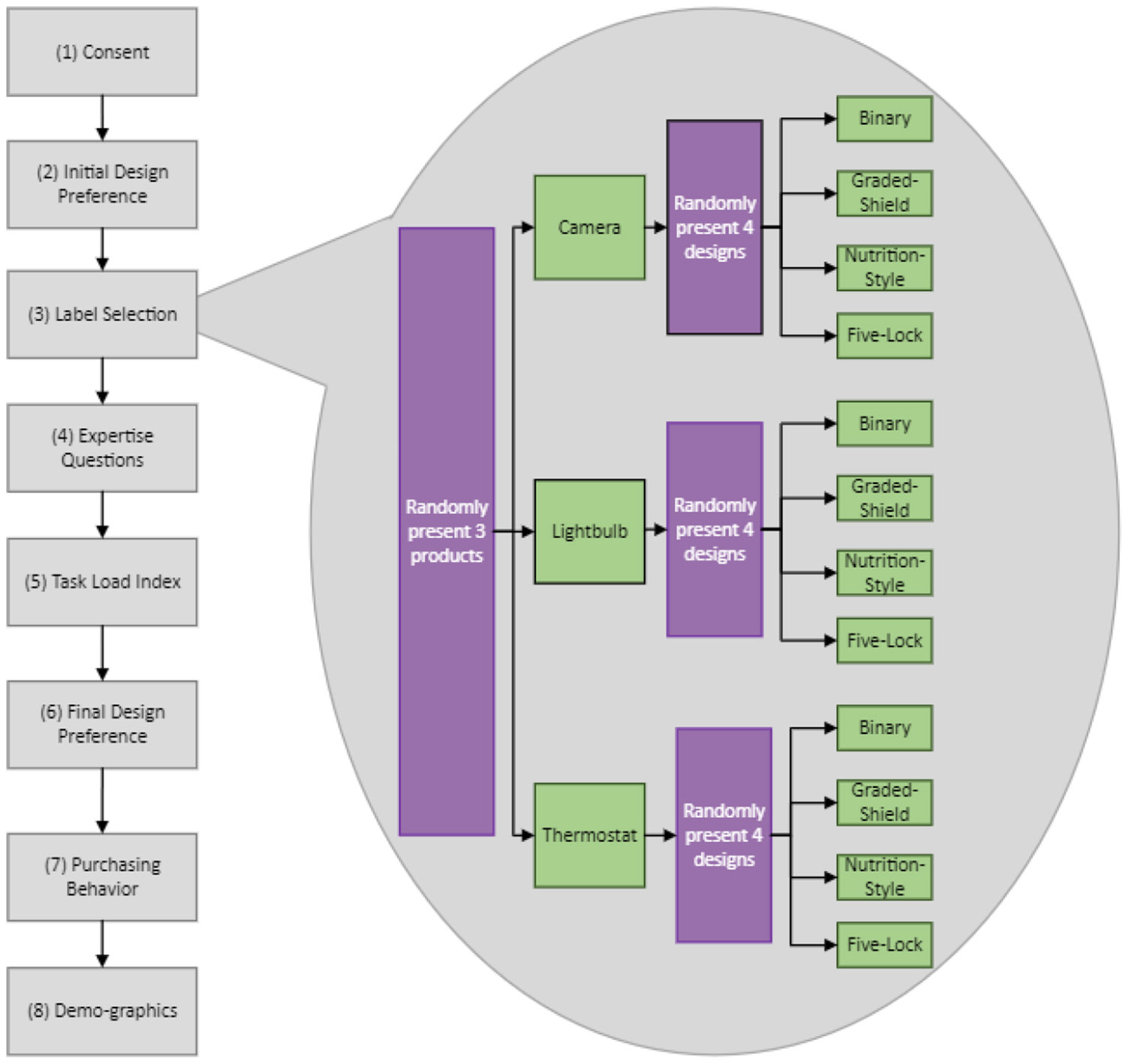
The process flow for the experiment asked participants questions across eight phases. The median time to complete was 00:17:14. In Phase 3, we randomized multiple parts of the study to distribute any potential bias across participants. The randomization is shown in detail in the callout bubble, as well as in the study protocol in Appendix B.7.

**Figure 10:**
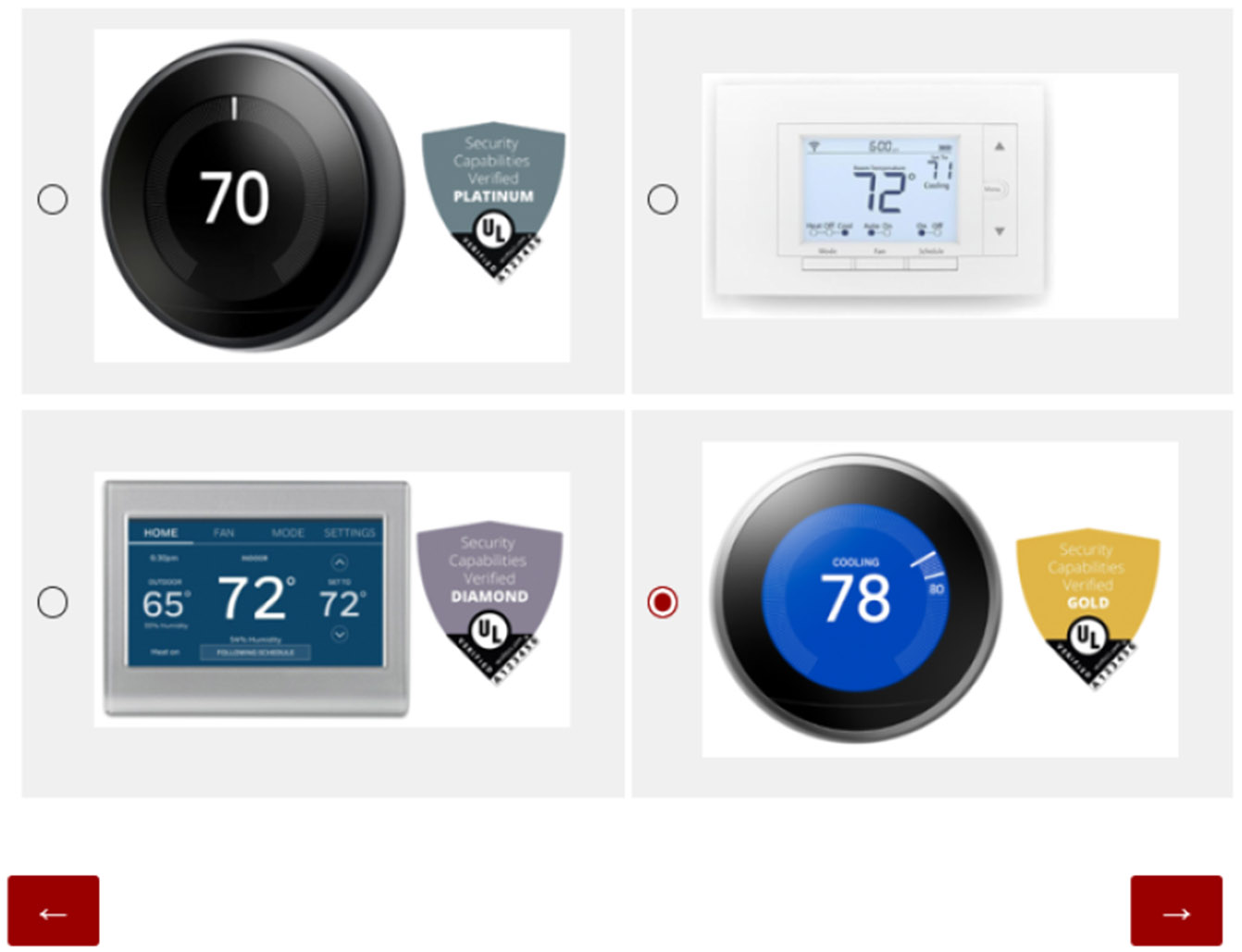
In Phase 3 of the study, participants were presented with four options and asked to select the one offering the greatest cybersecurity.

1. Phase 1 began by providing details about the research, including its purpose, procedures, potential risks and benefits, contact information, and participant rights.
2. In Phase 2, we asked participants to select the product they thought was most secure. We showed them four pictures of IoT cameras, each using a different security label design as shown in Figure 8. Cameras were used in tandem with labels, as we did not want to overtly influence the importance of labels at this time. All label designs were generated to represent a medium security quality and were randomly assigned to the cameras. For each participant, the answer order was randomized to avoid bias in the order of presentation. The goal for this phase was to establish what participants identified as the best indicator of security and why. See Appendix B.1 for the protocol for this phase.
3. Phase 3 of the study involved three rounds of product selection. Each round focused on different product categories: light bulbs, cameras, and thermostats. For each round, we asked participants to select the most secure product using a different label design each time. Figure 10 represents one product round using Graded-Shield labels conducted during Phase 3. Participants would have used each label design 3 times, selecting a total of 12 products during this phase. As we will discuss later in Section 6.3, some participants used knowledge of Graded-Shield labels to make educated guesses for later-presented Binary labels. Other participants, asserted certain product features were associated with more secure devices. Phase 3 has the potential for high levels of bias, so we took the following steps to minimize it.
  - To prevent unconscious learning bias, where prior information/selection could influence subsequent decisions, we randomly present each round of products. For example, Participant_One might be shown the order of light bulbs, cameras, and thermostats; Participant_Two might be shown the order of thermostats, light bulbs, and cameras.
  - For each round, we randomize the security quality of each device to minimize the impact of any unintended association or learning. Labels were applied according to device security. For example, in the thermostat round, the most secure device for the Nutrition-Style Label was the least secure for the Binary Label.
  - To minimize the influence of design bias, we use three products to aggregate results for an overall measure of influence. For example, we could not account for individuals interpreting certain camera aesthetics as being more secure; however, these attributes would not be present in a thermostat or light bulb. Aggregating responses across all three products will better represent label design efficacy.
  - The answer order was also randomized, as to avoid position bias, where participants may subconsciously favor the first choice. For example, Participant_One might be presented the devices in order 1,2,3,4 (in ascending order of security quality); Participant_Two might be shown them in order 2,4,3,1.
  By randomizing multiple parts of the study, we distribute any potential biases evenly across participants, making the study results more robust and reliable. The goal for this phase of the study was to ascertain the effectiveness of labels, as well as to familiarize the participants with the different label designs through their usage. See Appendix B.2 for the protocol for this phase.
4. In Phase 4 we asked each participant five questions to measure knowledge and expertise in the security domain. We asked participants to select the correct definition of data anonymization, brute force protection, secure onboarding, data retention, and data confidentiality. These concepts were salient because they are included in the proposed label designs, based on the CMU nutrition label [17], NIST proposed guidelines for security for IoT [49], the UL Label [64], security best practices [43], and the IEEE Building Guide for the IoT [39]. However, technical literacy was not a requirement for this study. The goal for this phase was to evaluate consumer’s understanding of the attributes being ensured by labels. See Appendix B.3 for the list of questions.
5. In Phase 5 of the study, we asked participants about their overall understanding and use of the labels. We use the National Aeronautics and Space Administration Task Load Index (NASA-TLX) to measure the perceived workload of each label across five dimensions: mental demand, temporal demand, performance, effort, and frustration [29, 30]. The goal of this phase was to determine the cognitive demands of each label design. See Appendix B.4 for the protocol for this phase.
6. In Phase 6, we explicitly ask about label design preference. Listing all four designs, we asked participants to select the label design that they preferred and why. The goal of this phase was to see if label preferences changed after having used the different label designs. See Appendix B.5 for the protocol for this phase.
7. The Phase 7 part of the study focused on purchasing behavior questions. Here we collect information on how participants typically evaluate and research products before purchase and any QR code habits. The goal here was to see how important security was to overall purchase decisions, as well as the usefulness of QR codes in enabling more secure procurement. See Appendix B.6 for the protocol for this phase.
8. Finally, in Phase 8 we collected demographic information, which we describe below.

### 5.3 Demographics

We recruited 500 participants via Prolific and collected demographic information on income, education, age, gender, race, and language. Prolific provided a representative sample across age, gender, and race for the United States. We used this distribution so that the observations and subsequent conclusions were more likely to be generally applicable when compared with a convenience sample or other less representative sets of participants.

Our study stipulated that all participants must be at least 18 years or older and reside in the United States. Like the U.S. population, our sample was primarily white and middle-aged. The mean age of our respondents was 45.8 years old, and the median was 46 years old. Because of the literacy and computer access requirements, our population was relatively educated (59% of participants have a bachelor’s degree or higher), with fewer high school graduates, compared to US census data. Income among our participants was lower than the US household median income of $74,580, with only 37% having an income greater than $60,000.

A label that is designed for an average consumer will exclude vulnerable segments of the population. Further threads of this research will be explored later in Section 7. But at a high level, in order to mitigate this, when there is a highly variable audience effective labels are those designed for the least expert [70]. This diversity reifies the importance of crafting inclusive labeling strategies, taking into account different backgrounds, expertise, and cultures.

## 6 RESULTS

In this section, we report on the effectiveness, perception, and preferences of security label designs. In doing so, we can better understand which design principles can create a more secure ecosystem for consumers.

### 6.1 Effectiveness of Security Labels

At their core, labels are designed to help consumers make more informed decisions. If they fail to do this, they are not effective. We define label effectiveness by the accuracy of the most secure selection, the speed at which they can make it, and the perceived assurances of a given label. We will also look at participant QR code utilization and if it is feasible to use a secondary label to communicate future information to consumers.

#### 6.1.1 Accuracy.

In order to impartially evaluate the label designs’ effectiveness, we measure participants’ ability to select the most secure product. To do this we present participants with four different options for a product, mimicking a real-life camera, light bulb, or thermostat. We removed brand identifiers. We assigned security and privacy attributes to each product and attached an associated label from the design. The results in Table 2 show that users were able to overwhelmingly identify the most secure product using the Five-Lock label with an average accuracy across all products of 88.03%. Both the Graded-Shield and Nutrition-Style labels performed well, with an average accuracy of 46.03% and 52.23%, respectively. The low accuracy for secure Nutrition-Style label in the *light bulb round* brought down the average; however, the *thermostat round* shows that participants were able to be highly effective using this label, at 65.2%. The Binary label did no better than random chance, with an average accuracy across all products being 21.23%.

We utilized a one-way analysis of variance (ANOVA) to evaluate if there are statistical differences in accuracy across different labels for three different products. The ANOVA results suggest that the labels’ accuracy differ significantly, as the camera results in F(3,1593)=129.27, p<.001, the thermostat results in F(3,1593)=209.16, p<.001, and the light bulb results in F(3,1592)=173.59, p<.001. When the ANOVA outputs indicate there is a significant difference between the means of the groups, it is important to follow up with a post-hoc test to determine which specific groups are driving the overall difference. For this, we use the Tukey Honestly Significant Difference (HSD) to conduct pairwise comparisons of group means, as it provides a more conservative approach to control the family-wise error rate.

The Tukey HSD results, listed in Table 3, reveal significance values of <.001. This statistical analysis indicates that the observed differences between these labels are highly unlikely to be a consequence of random chance alone; they indicate a statistically significant dissimilarity. Like any statistical method, it is important to note that while our findings are statistically significant, further study is required to ascertain the practical robustness of the results. Still, this level of significance shows that different label designs do influence consumer accuracy. Therefore, we reject the null hypothesis and infer that there is a substantial and meaningful distinction in accuracy between the different label designs.

#### 6.1.2 Timeliness.

**Figure 12:**
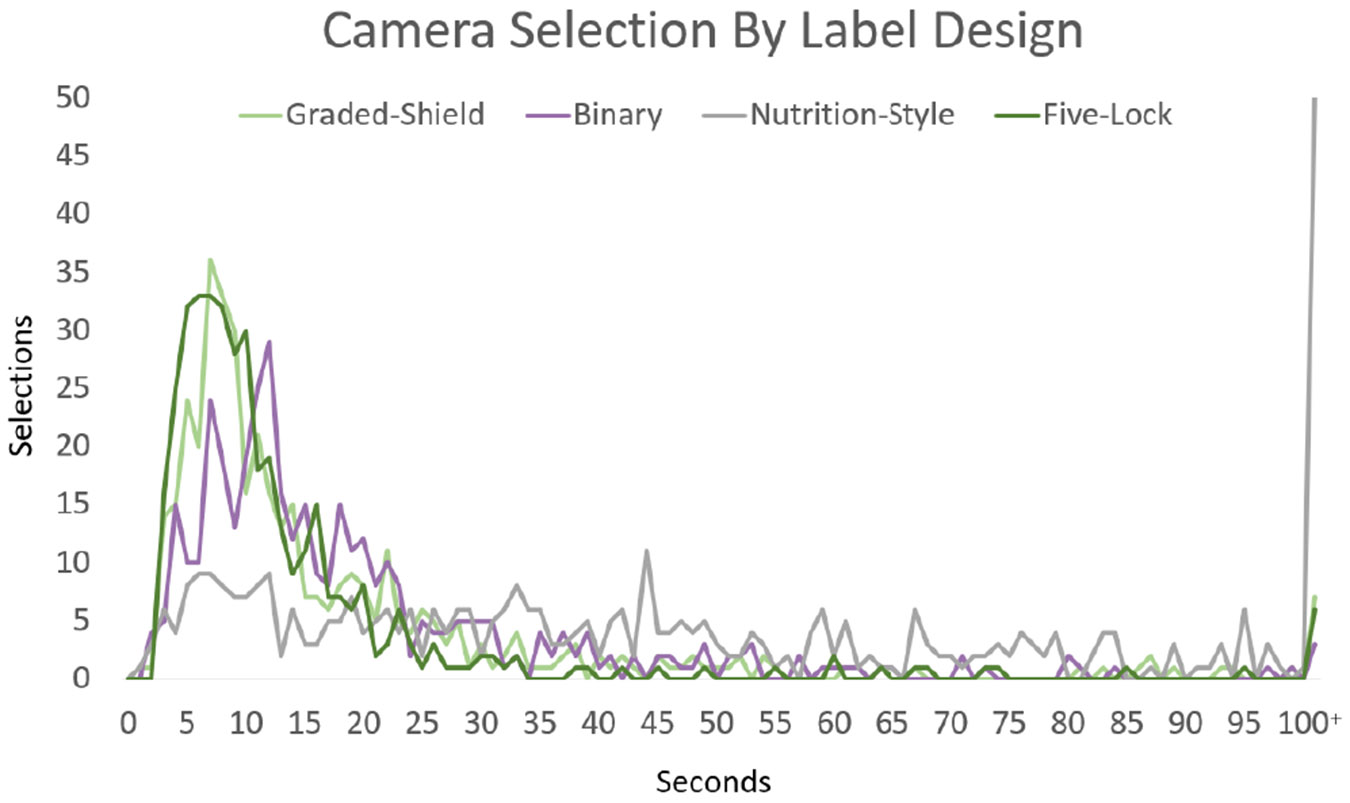
Time to selection across all four label designs. After 100 seconds, responses are grouped. This trend is reflected in both Thermostats and Light bulbs, whose charts are located in Appendix A.

**Figure 13:**
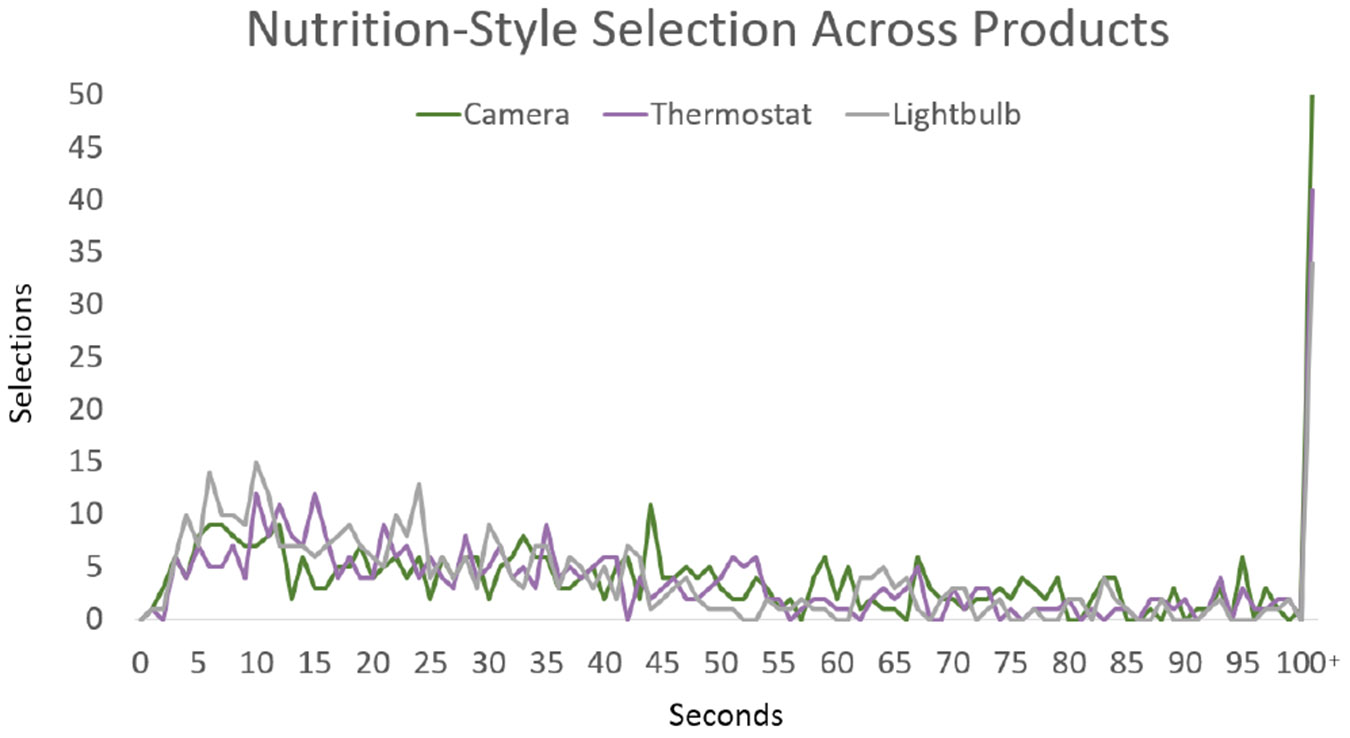
Time to selection aross Nutrition-Style labels took longer to use and are the only label design with statistically different selection times. Additional charts for the other label designs are located in Appendix A.

Not only should a label support accurate decisions, but it should provide information in a timely manner. Shorter times are indicative of a label that may be more efficient and user-friendly. If the presented information is clear and comprehensible, it enables users to quickly assess the security quality to make decisions. To measure the timeliness of labels in aiding decisions, we measured page submission times. Doing so allows us a more quantitative look at how cognitively burdensome the label designs may be. Looking across response times, we can see that both types of graded labels (i.e., Graded-Shield at 19.8 seconds and Five-Lock at 19.0 seconds) had the quickest time to decision. However, the median for the Five-Lock label was the lowest at 9.9 seconds, while the standard deviation was 56.28 seconds, one of the highest; this is likely due to the use of QR codes taking participants to secondary labels. This still demonstrates that an effective label design not only influences more accurate decisions but will be able to do so quickly. The Nutrition-Style label, the other label that performed well in our accuracy test, had the longest time to decisions, with a mean of 47.94 and a median of 32.4 seconds. While it provides the most information at a glance, it does so in a manner that requires consumers to read each label and independently ascertain security levels. Finally, the Binary label performed well in this study with an average response time of 22.3 seconds, but likely security decisions were based on other factors besides the label designation. The outputs from this timing study are shown in Figure12 and Figure 13. When labels do not impose a significant cognitive burden, consumers can compare across products more easily. This leads to improved user experiences, increased task efficiency, and more effective purchasing decisions.

We again utilized one-way ANOVAs to evaluate the statistical significance of the labels’ effect on time-to-decision. The ANOVA results suggest that the labels’ timing differs significantly, as the camera results in F(3,1593)=63.94, p<.001, the thermostat results in F(3,1592)=19.51, p<.001, and the light bulb results in F(3,1592)=18.92, p<.001. The Tukey HSD results, listed in Table 4, reveal p-values of <.001 for each label design compared to the Nutrition-Style labels. This statistical analysis indicates a statistically significant dissimilarity between time-to-decision for Nutrition-Style labels vs. the other three designs. Regarding the other three labels (i.e., Binary, Graded-Shield, and Five-Lock), the Tukey HSD significance values reveal no significant difference between their timing. Therefore, we can infer that there is no distinction in timeliness between the Binary, Graded-Shield, or Five-Lock label designs; however, there is a substantial and meaningful distinction in the longer times for Nutrition-Style labels.

#### 6.1.3 Assurance.

Additionally, the label should provide assurances of specific security traits. To do this we asked participants if they felt that their selected product provided security features and ensured specific security concepts. We did not inform the participants about the underlying requirements to earn each label or grade. Additionally, we assume they have no prior knowledge of this information. As two of the labels lacked any information claiming specific assurances, the consumer would rely on the organization or the quality of the design to ensure it. The lack of transparency is evident with significant uncertainty across all label designs. Even the Nutrition-Style label, with the most information available at a glance, was not able to provide high levels of assurance. In Table 5 we report the results of one round of our study (i.e., camera round). We can see a trend occurring where users who are able to see more information are more willing to trust the assurances of other qualities, even in the absence of them being explicitly labeled.

As this will be deployed to the general population, we did not pre-screen for technical knowledge or skills; instead, within our survey we asked for familiarity with basic security concepts. Participants were asked to select the correct definition of five security factors that were included in the labels or ratings. Fewer than half understood secure onboarding (48.1%), and barely a third could correctly identify the definition of brute force protection (34.0%). In contrast, participants had a much better understanding of those variables relating to data practices, with 73.6% understanding data confidentiality, 75.1% correctly defining data anonymization, and 62.2% selecting the correct definition of data retention.

#### 6.1.4 QR Codes.

Secondary layers are especially beneficial to consumers who are making more technical or higher-level integration decisions. However, prior research has shown a low utilization of QR codes [6, 33]. To test this, we hosted working secondary layers, linked through QR codes on a separate webpage, and tracked view counts. Across the study, the total number of QR codes scanned and followed was only 42. Each participant was exposed to QR codes 24 times, meaning that 99.65% of the time our participants did not use the provided QR code for more information.

At the conclusion of our label study, we asked QR code utilization questions. Prior to study access, we validated all participants would take this survey from a laptop or desktop, to ensure that they could scan QR codes if they desired. However, we do not prime them on the function or use of QR codes in the study. At the end of the study, we asked participants if they had a QR reader; 93.6% of our respondents knew they had a phone with a built-in QR code reader and would have this capability during our study. However, 84.9% report only rarely using QR codes, monthly or less. One specific question asked if the participant used any of the QR codes in this study. Of the 500, 45 responded that they did use a QR code; however, even if each one only scanned one QR code through the entire study, we only captured 42 page views. This discrepancy could be explained by how the participant defines “use” or by a difference between self-perception and action. QR code readers have the ability to scan and check the link before following. It is possible that participants checked the QR code to see if it was a valid link but did not follow it to the secondary layer. When we asked, in practice, if they would use a security label’s QR code to gather more information on a product, only 9.9% said they definitely would. This is larger than the 8.4% that corresponds to 42 users and is close to the 9% that asserted use in the experiment.

### 6.2 Perceptions of Security Labels

The usefulness of labels is also contingent on the cognitive burden the label places on the individual. In the previous section, we measured this mental load by measuring the time to selection. Designs that allowed quicker time to selection were thought to be less mentally taxing. In this section, we will use NASA-TLX questions and a 20-point scale to measure the cognitive demands across mental, temporal, performance, effort, and frustration dimensions [29, 30]. The Binary label, which is touted for its simplicity, was not found to be easier to use. Instead, it was seen as the most frustrating and required the second-most effort and mental demands to use. The Nutrition-Style label had the highest mental demands, took the longest time to use, and the most effort, but it resulted in participants believing they had a more accurate selection. It also accurately provides information that many participants simply assumed was embedded in other labels, as noted above. The Five-Lock label performed the best across all TLX measures.

When asked about other factors that influence procurement decisions, participants ranked these factors as most influential: (1) price, (2) privacy, (3) cybersecurity, (4) brand, (5) other. While security is important, it does not emerge as the primary driver behind purchasing decisions. Yet, given the importance of security and privacy, this validates the development of a security label, demonstrating both the importance and the lack of primacy. Labels can address security and privacy with brevity and clarity, supporting a consumer’s multidimensional decision. The lack of primacy also suggests that such labels should not impose an undue cognitive burden in an effort to be more valuable. Because while it is valuable, an excess of irrelevant information has the potential to obscure the overall decision-making process, to the point where the security label is not even utilized.

### 6.3 Security Label Preferences

Prior to conducting the main rounds of the label study, and without explanation of the label designs, we asked participants to select an IoT Camera that provided the highest level of security. To do this we presented four IoT cameras using the four label designs. The label grades represented a medium security posture and were randomly assigned to the IoT cameras. Not only did we capture selections, but also initial responses for their decision-making. These responses allowed us to understand participant label preference, or if they were evaluating security on a different basis (e.g., camera design features). At the conclusion of the study, we directly asked which label they preferred as a way to measure the change in perception of usability over time. At the end of the study, the majority of respondents, 46.3%, felt like the Five-Lock label design was able to succinctly communicate security posture. The other three label designs saw a significant drop in perceived utility. The post-study survey revealed the Binary label and Graded-Shield label were seen as too superficial to add value to product decisions; while the Nutrition-Style label was too dense to be able to support decisions at the time of purchase. We address these changes in future work.

We assessed the statistical significance of label preference changes between the pre and post-experiment using McNemar’s test, which allowed us to determine if there was a significant difference in the distribution after label use during the study. Based on the resulting significance of <.001, we can reject the null hypothesis: that there would be no significant difference between the pre and post-experiment. This shows there is a statistically significant difference in the label design selection before and after the use of the design. The findings suggest that the use of the label had a significant impact on the resulting change in preference.

### 6.4 Qualitative Coding

We also gathered thematic reasoning for each label design, pre and post-study. To do this, when selecting the label in the pre-study, we asked our participants to also justify their selection. We then used a structured codebook aimed at understanding and documenting underlying themes within these responses. Once we had coded the responses, we were able to synthesize insights into the participants’ reasoning and preferences for specific design features as indicators of security. The qualitative analysis illuminated several key patterns and trends, which led to underlying cognitive processes to ascertain security among products. These insights can inform future designs.

In the pre-study, 38% of participants did not leverage labels as an indicator of security quality. Instead, their decisions were predominantly shaped by a multifaceted amalgamation of factors encompassing the camera’s visual aesthetics and perceived functional attributes. In many instances, the absence of functional information regarding the cameras (e.g., price, storage, Wi-Fi, IR) drove participants to rely upon intuitive judgments and personal preferences in determining the security quality. However, it is noteworthy that specific label designs were more readily accepted as providing security assurances. Nutrition-Style labels, while not well understood by participants (e.g., they had a severe aversion to the Camera being manufactured in China), were seen to provide security assurances only by virtue of being the most verbose. On the other end of the spectrum, participants manifested a high preference for “Gold” and “Verified” offered by the Graded-Shield as being both the highest guarantees of security and privacy.

Both the Graded-Shield and Binary labels were appreciated for their label simplicity and their design aesthetics (e.g., color and shape). The Graded-Shield label maintains higher trust due to the verified security claims; however, the gradation of the design does not seem to communicate as it intends, often causing some levels of confusion. Nutrition-Style labels maintain their usability by virtue of information conveyance, but to fully be utilized, it would need the participant to have more technical understanding. Participants noted the significant effort to compare across products, often honing in one aspect of this label as a point of difference (e.g., the manufacturer location). In this regard, the label was seen as simpler to use, while the additional information made it more robust. The Five-Lock label saw the greatest jump in usability due to the intuitive design of an internal grading scale. In the following section, we highlight more takeaways of each label design.

#### 6.4.1 Binary Label.

In our pre-study, the majority of the selection decisions bearing the Binary label centered around product design. Most participants acknowledged tendencies to either select randomly or base choices on the product’s visual aesthetics. Product dependencies were the result of the randomization and allocation of labels to products. During the study, due to the lack of information, some participants established a cognitive link between the current product and previously questioned products. Post-experiment, participants considered the mark bearing assurances from the U.S. and valued the aesthetics of the label design (particularly the color).

#### 6.4.2 Graded-Shield Label.

The Graded-Shield label saw the largest drop in usability. Participants in both pre and post-study found that the label from a recognized vendor carried more weight than the other labels. However, in the pre-study some participants assumed that “gold” was the highest guarantee. Without an internal scale, consumers gravitated toward gold being the highest assurance of security compared to the other three designs (even though they all represented medium security quality). This significantly inflated these earlier results. During use, participants recognized that the diamond denoted a higher level of security; however, this was not a universal consensus as seen by accuracy results, averaging around 46.0%. Still, post-study participants were familiar with this labeling scheme, and they felt like this label still adequately supported decision-making, while also noting that the seal was verified.

#### 6.4.3 Nutrition-Style Label.

Pre-study, the Nutrition-Style label was seen as the highest indicator of security. Participants noted that transparent security and privacy specifications allowed for a more detailed understanding of the particular product. This would help ensure future comparisons would be based on this information; however, post-experiment, participants found that the detailed information was valuable but harder to compare between products when it was not fully disclosed. They appreciated the first layer of information as it was perceived as a sign of manufacturer transparency. However, participants would gravitate towards one security indicator as providing overall security. For example, participants noted a product being made in the USA as an indicator of higher security. This could be one reason for reduced accuracy in the Light bulb round, where all the products originated from the USA.

#### 6.4.4 Five-Lock Label.

The Five-Lock label was seen as providing the lowest security assurance during the pre-study. The label used an internal grading scale (i.e., the label we used scored 3 out of 5 locks), and participants assumed that this meant it was less secure compared to the others (e.g., gold). Participants also noted that the particular camera design looked less sophisticated, though this was an artifact of the study design and randomization. Post-study, this label saw the most gain, with 43.1% more users finding it to be the best indicator. For use, this label was seen as the most straightforward, enabling consumers to quickly assess security levels across products. Some participants also noted that the inclusion of the QR code led to more security information on particular products, which validated the scores.

## 7 DISCUSSION AND IMPLICATIONS

The results of this research can inform the development of more secure and consumer-friendly labels; labels that support US consumers in making choices that best align with their risk tolerances or security preferences. Security label design should enable consumers to make more accurate, timely choices without overburdening them. Labels ideally would communicate security information efficiently at the time of purchase. Our survey showed that 51.9% of participants do not do prior research on a product before purchasing. That implies security information should be available on the product at the point of purchase to best inform decisions. Security is just one aspect that influences purchasing decisions.

Even after being required to evaluate products on the basis of security, participants ranked privacy as more important than security in procurement decisions. Thus, effective security label designs could be more salient if these included privacy factors as well as security. Security and privacy concepts are highly intertwined. If privacy is the goal then security is that enabler. It is critical to not only design security in a way that protects consumers, but it is equally important to communicate in a way that reflects consumers’ preferences.

The change in label preference after use indicates that label preferences will inevitably evolve with use. Thus, security label design can be seen as a process, where the U.S. Cyber Trust Mark may be a first step, not a final design. Labels should support current consumer decisions but also be able to expand with future needs and changing preferences. A security label that does not provide relevant information is not valuable. While participants noted a preference for simpler designs, a significant portion found value in having more information readily available. Even if the consumer does not intend to use a label, the existence of the Nutrition-Style label communicates a higher degree of security assurance. For a voluntary label program, this may be preferable.

However, an important implication of our experiment is that simply providing more information does not necessarily mean more informed or more secure purchasing decisions. Additional information can overwhelm users, leading to increased cognitive load and prolonged decision-making times [41]. The result is information overload, where too much information prevents consumers from evaluating relevant data to make an informed decision. This insight challenges the assumption that providing consumers with more label information is better [3, 25, 33, 46]. The obvious solution may not be so simple as just moving this information to a secondary layer; QR codes, which were leveraged by two of the label designs, were not highly utilized by study participants. Using a simple binary label ensures compliance, but a graded label can communicate both compliance and note the products that are better than merely compliant. This underscores the need for a balance between conveying essential security information and ensuring that labels remain user-friendly and comprehensible.

The four different label designs demonstrate ongoing, disparate efforts employed to solve the same problem. Unification is essential to enhancing consumer trust and legitimacy. Certain attributes of label designs are better for certain tasks and are contingent on the purpose of the user (e.g., a technical user wants lots of information readily available). Our results show that the Five-Lock label performed well across tests; however, one design is not inherently better for all situations. Still, standardization of any security labeling scheme will enable vulnerable consumers to make informed choices. As security is only one dimension of a multi-dimensional decision-making process, designers need to be cognizant of the limited amount of physical and mental space a security label can impose.

## 8 LIMITATIONS

We must consider some limitations of our study and the potential generalizability of our findings. We recruited 500 participants as a representative sample of current US demographics. However, due to the nature of Prolific, those without a high school degree or access to a computer are under-represented when compared with statistical analyses of the United States. The sample is representative only in terms of US gender, age, and race. It is also important to acknowledge that our study is context-specific to the US, and it may not be directly applicable to other countries with different regulatory frameworks or cultural contexts. The generalizability of our findings beyond the scope of the US should be interpreted with caution.

In our study design, we sought to cover a range of designs: binary, graded, descriptive, and multi-layered. We examined a specific design of each type. A more comprehensive assessment might compare different designs within a type. For example, a more aesthetically pleasing graded label might prove more acceptable, useful, and usable. Likewise, more developed descriptive labels (i.e., similar to the secondary layer of the Five-Lock label) may be more accessible. Finally, these designs just provide a cursory coverage of the range of possibilities and do not necessarily cover disadvantaged users such as those with limited language skills or technical acumen.

The presence of divergent product images featuring various security labels appears to have introduced a potentially confounding element, particularly when engaged in cross-genre comparisons of these security labels. Creating a survey that successfully avoids presenting identical imagery with different security labels poses a significant challenge, as participants might mistakenly perceive them as identical products. It is crucial to carefully choose survey product images that are different yet fall within the same range of perceived aesthetics and features. This would minimize any unintentional impact on participant decision-making attributed to disparities in aesthetics and features.

The survey experiment was intended to obtain participant preferences. However, participants did not make purchases. Individuals may express one preference yet act as if they had another. For example, participants regularly express a desire for health, but fewer people engage in consistently healthy behavior. Surveys are a critical but not comprehensive method for evaluating preferences and decision-making.

## 9 FUTURE WORK

This work evaluated the efficacy of four security label designs compared to each other as the primary point of difference. The labels selected were binary, graded, nutrition-style, and graded multi-layered. Even after selecting products based on security, security was not identified as the most important factor in evaluating IoT devices. This reifies previous work, which found that even when security is essential, it is just one of many considerations consumers make when selecting products [5]. Future work should consider the interplay of other factors (e.g. price, privacy, brand, features) to further evaluate the efficacy of security labels for different people, products, and contexts. Additionally, other form factors should be tested with consumer interaction, particularly in comparing labels on packaging and on physical shelves. An intuitive design that supports online purchasing may not be useful or usable in different modes of consumption. Previous research on consumer decision-making offers open questions with respect to the potential use of priming, product display, and label or display defaults. Finally, measures of both unconscious and purposeful engagement, such as with mouse tracking or eye-tracking, could inform the label’s ability to draw and hold attention.

The changes in preference for labels might vary considerably if the participants were spending money or if they were making only a single decision. The perceived burden of the Nutrition-Style label may not appear in studies where participants are spending money, as the salience of the information may be higher. Participants might become familiar with different labels and underlying assurances over time (e.g., learning that the gold Graded-Shield label is the third of five levels). Such familiarity could be provided with participant instruction and may improve the acceptability of that label design.

The QR codes were not used, and most participants did not express a desire to use them. However, the most strongly preferred labels before and after included QR codes. Thus, like warranties or instructions, the existence of the QR codes may be perceived as valuable even by those who do not use them. Future work could also explore the salience of QR codes, by comparing otherwise identical labels with and without codes.

This study focused on individuals within the United States; however, even with the locality constraint, our participants possessed a diverse set of language skills. Of our 500 participants, 70 were fluent in more than one language, and 22 had a primary language other than English. While unintended, this was seen as particularly valuable to our research since labels should be accessible to all users, even when English is not their primary language. While we did not collect enough data to make any definitive claims, it highlights the need for more extensive usability studies. Testing across a spectrum of user-profiles would allow us to validate the efficacy of a chosen labeling design in protecting the most vulnerable subsections of the population. By incorporating feedback from individuals representing varying levels of expertise, cognitive abilities, and cultural perspectives, labels can be more universally accessible. Future work should look at working with disadvantaged populations, and testing labels that focus on multilingual and/or adaptive displays.

## 10 CONCLUSION

In this work, we compared four categories of labels. Upon initial exposure, participants found the descriptive multi-layer label (i.e., the Nutrition-Style label) to best suit their needs. After completing the study by evaluating three products, the participants shifted their preference toward the graded multi-layer label (i.e., the Five-Lock label). While both of these labels provide QR codes, they were rarely used and only a few participants expressed an interest in using them. Participants preferred simple indicators that clearly delineate the security of products. Labels that lacked these noticeable differences were perceived as difficult to use, and participant accuracy was low (i.e., the Binary label). Overall, the concept of graded labels was seen as a useful indicator to ensure security; however, the lack of an intuitive or understandable scale made it difficult for participants to leverage (i.e., the Graded-Shield label).

This study emphasizes the importance of understanding the underlying perceptions, motivations, and behaviors that shape individuals’ responses to security labels. It goes beyond simple design comparisons by examining the cognitive workload and accuracy of decision-making. In doing so, we address not only the content of the label but also how participants interpret and use that information in their decision-making process. It underscores that security and privacy are multifaceted concerns. Our evaluation addressed cognitive load, comprehension, and acceptability. The insights gained from this study can inform the ongoing label discourse in the US, with the goal of creating designs to enable consumers to easily and accurately make more informed decisions.

## Figures and Tables

**Figure 11:**
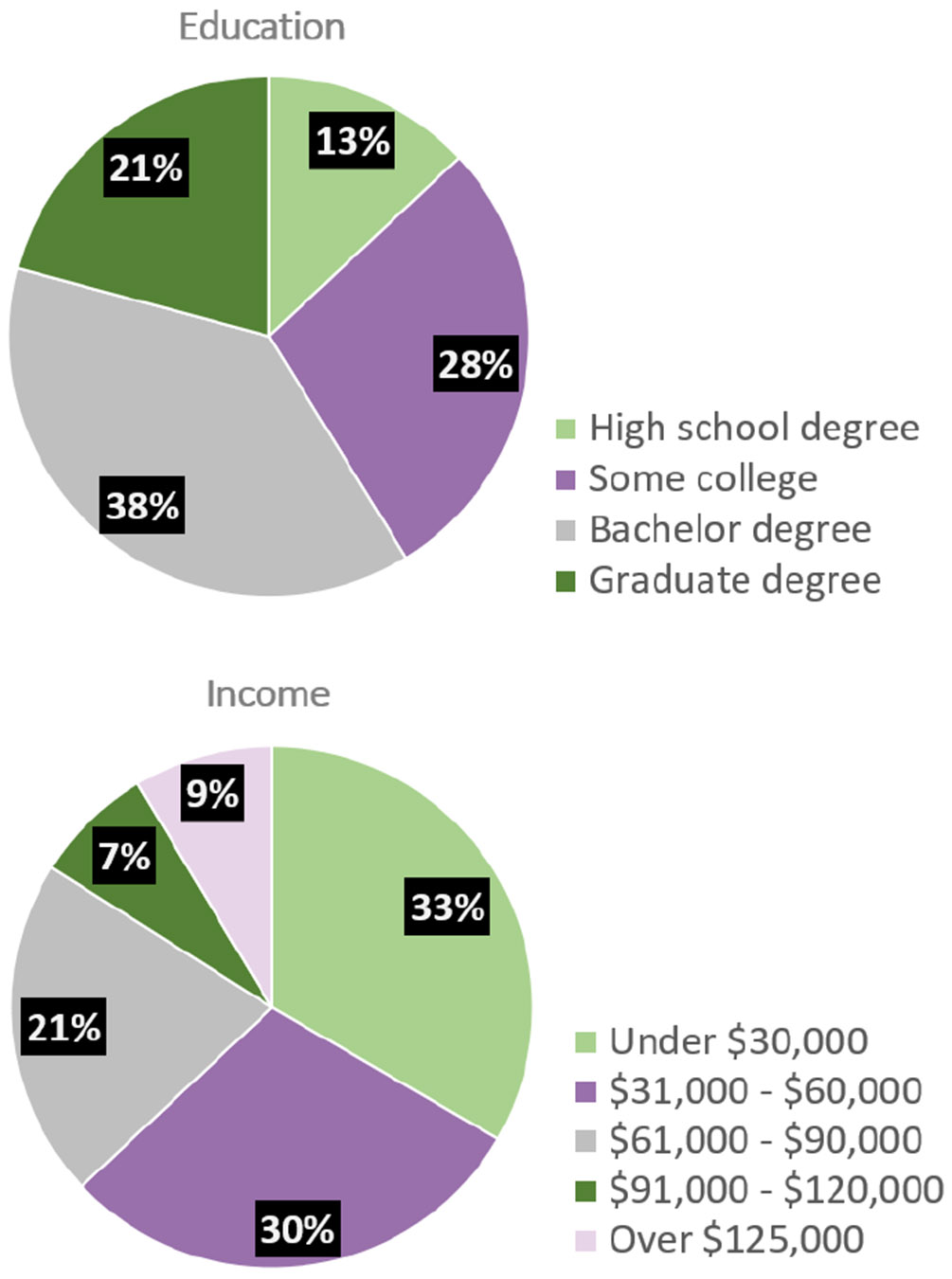
Demographic distribution of the 500 participants with respect to education level and annual income.

**Figure 14:**
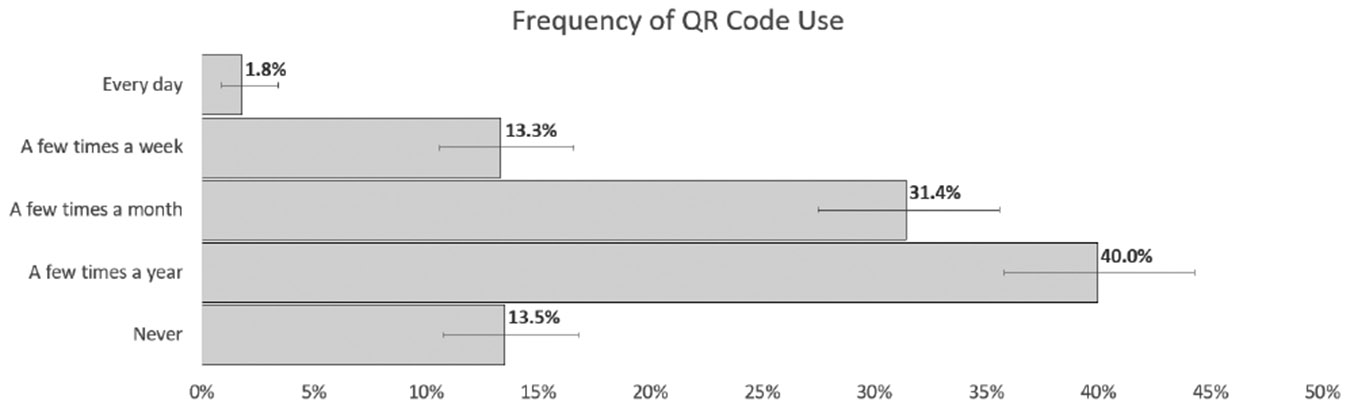
Responses for frequency of QR code use in everyday life show that most only use QR codes a few times a year.

**Figure 15:**
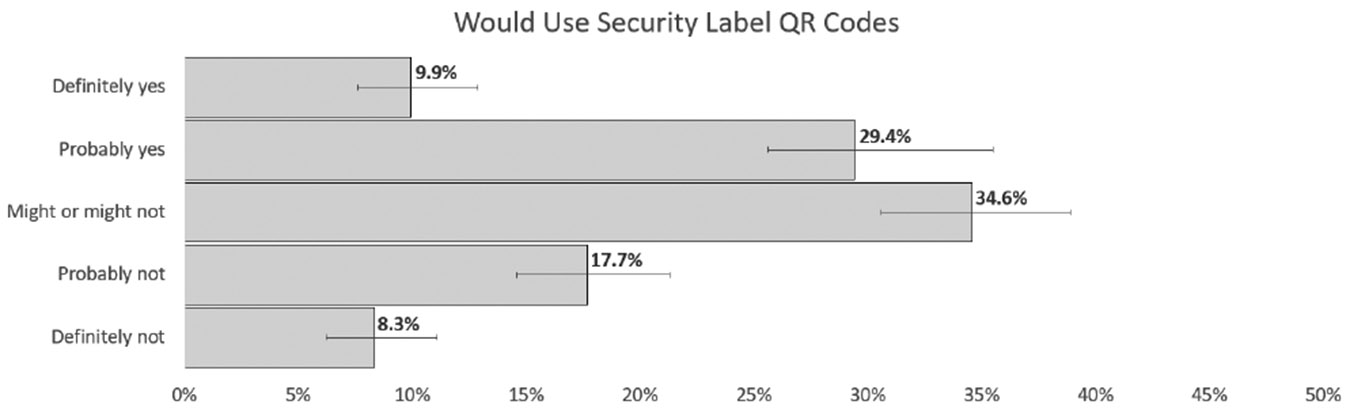
Only 39.3% said that they would use QR codes to better inform decisions while 26% said they would not.

**Figure 16:**
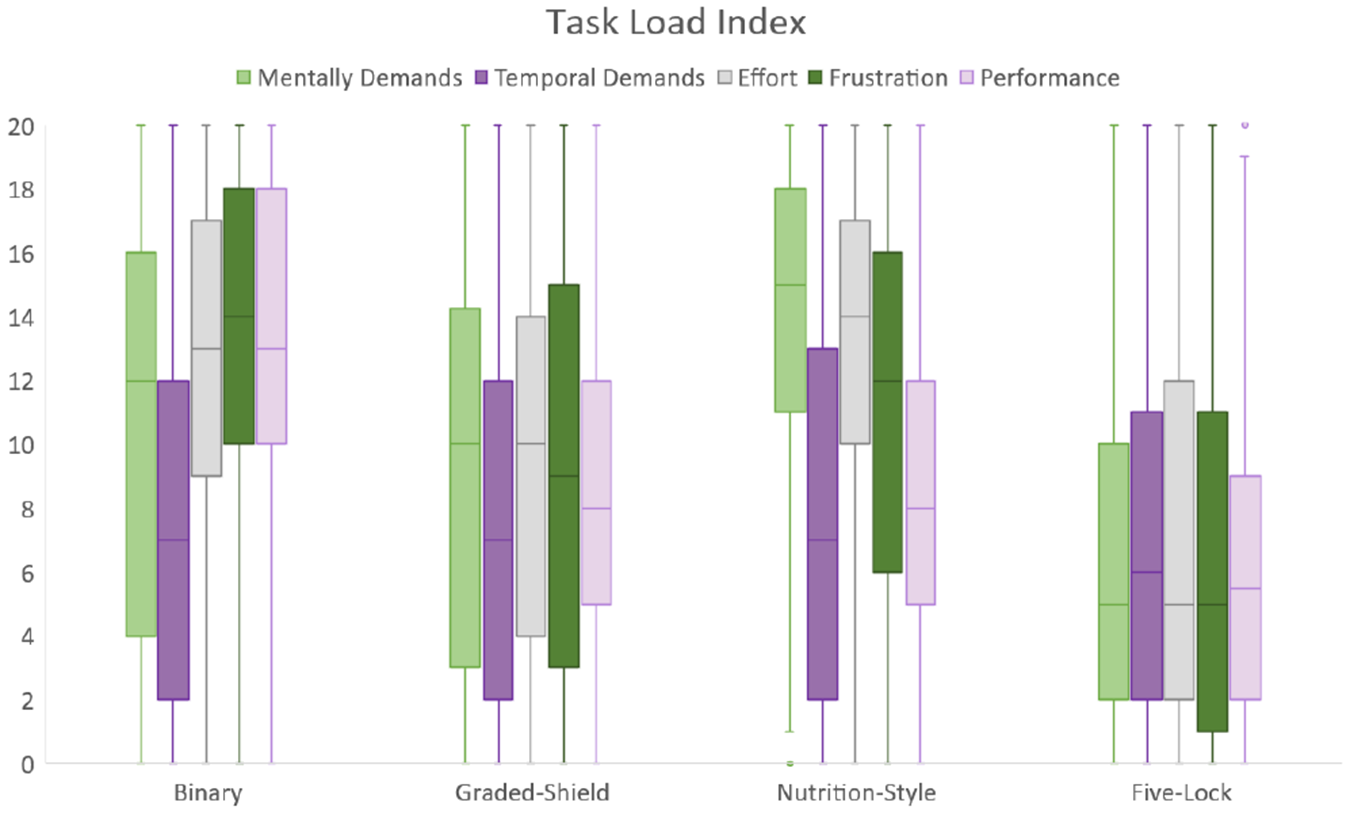
Graphical representation of the TLX, showing the distribution of responses. The lower the score, the less mentally demanding the participant believed the label to be.

**Table 1:** The distribution of this representative sample per three demographic factors with study participants approximating the US national population.

Participant Demographics						
Gender/Race	Age					Total
	18-27	28-37	38-47	48-57	58+	
**Female**	**44**	**37**	**48**	**44**	**83**	**256**
Asian	3	3	3	3	4	**16**
Black	7	6	6	6	9	**34**
Mixed	2	0	2	1	1	**6**
Other	1	2	1	1	0	**5**
White	31	26	36	31	69	**195**
**Male**	**43**	**39**	**44**	**41**	**72**	**239**
Asian	3	2	4	2	3	**14**
Black	6	6	5	5	7	**29**
Mixed	2	1	1	1	1	**6**
Other	1	1	1	1	1	**5**
White	31	29	33	32	60	**185**
**Self-Described**	**3**	**1**	**0**	**0**	**1**	**5**
Black	1	0	0	0	0	**1**
Mixed	0	0	0	0	1	**1**
White	2	1	0	0	0	**3**
**Total**	**90**	**77**	**92**	**85**	**156**	**500**

**Table 2:** Selection accuracy by smart device.

Security Accuracy				
	Camera	Thermostat	Light Bulb	Avg
Binary	28.1%	14.2%	21.4%	21.2%
Graded-Shield	45.4%	48.9%	43.8%	46.0%
Nutrition-Style	56.7%	65.2%	34.8%	52.2%
Five-Lock	**89.0%**	**87.3%**	**87.8%**	**88.0%**

**Table 3:** The results of the Tukey HSD post-hoc tests show that accuracy between all label designs is <.001. This indicates that there is strong statistical evidence to suggest that the labels’ accuracy is significantly different among the various designs and is unlikely to have occurred by random chance.

Camera Accuracy				
	Binary	Graded- Shield	Nutrition- Style	Five-Lock
Binary		**<.001**	**<.001**	**<.001**
Graded-Shield	**<.001**		**<.001**	**<.001**
Nutrition-Style	**<.001**	**<.001**		**<.001**
Five-Lock	**<.001**	**<.001**	**<.001**	
Thermostat Accuracy				
	Binary	Graded- Shield	Nutrition- Style	Five-Lock
Binary		**<.001**	**<.001**	**<.001**
Graded-Shield	**<.001**		**<.001**	**<.001**
Nutrition-Style	**<.001**	**<.001**		**<.001**
Five-Lock	**<.001**	**<.001**	**<.001**	
Light Bulb Accuracy				
	Binary	Graded- Shield	Nutrition- Style	Five-Lock
Binary		**<.001**	**<.001**	**<.001**
Graded-Shield	**<.001**		**<.001**	**<.001**
Nutrition-Style	**<.001**	**<.001**		**<.001**
Five-Lock	**<.001**	**<.001**	**<.001**	

**Table 4:** The results show significant variability between the Nutrition-Style labels and the other three labeling designs (i.e., Binary, Graded-Shield, Five-Lock).

Camera Timing				
	Binary	Graded- Shield	Nutrition- Style	Five-Lock
Binary		0.972	**<.001**	0.936
Graded-Shield	0.972		**<.001**	0.738
Nutrition-Style	**<.001**	**<.001**		**<.001**
Five-Lock	0.936	0.738	**<.001**	
Thermostat Timing				
	Binary	Graded- Shield	Nutrition- Style	Five-Lock
Binary		0.765	**<.001**	0.903
Graded-Shield	0.765		**<.001**	0.991
Nutrition-Style	**<.001**	**<.001**		**<.001**
Five-Lock	0.903	0.991	**<.001**	
Light Bulb Timing				
	Binary	Graded- Shield	Nutrition- Style	Five-Lock
Binary		0.391	**<.001**	0.837
Graded-Sheild	0.391		**<.001**	0.076
Nutrition-Style	**<.001**	**<.001**		**<.001**
Five-Lock	0.837	0.076	**<.001**	

**Table 5:** Percent of respondents that believe the label assures certain security factors for Smart Cameras. Only those using the Nutrition-Style label or those utilizing the QR code on the Five-Lock label should have any assurance of a particular feature. Thus while they may be correct, there was no basis for this confidence.

Assurance of Camera Security						
	Brute Force	Onboarding	Retention	Anonymization	Privacy	Confidentiality
Binary	20.7%	19.9%	21.2%	22.5%	26.6%	24.6%
Graded-Shield	23.8%	24.3%	25.1%	23.8%	35.7%	30.0%
Nutrition-Style	**33.1%**	**38.2%**	**45.7%**	**44.8%**	**57.9%**	**54.5%**
Five-Lock	28.7%	28.4%	26.9%	27.6%	36.7%	31.8%

**Table 6:** Captured number of page views show QR codes were only used 42 times during this study.

Secondary Label Utilization				
	Camera	Thermostat	Light Bulb	Total
Nutrition-Style	7	5	5	**17**
Five-Lock	13	7	5	**25**

**Table 7:** Average (mean) NASA-TLX measures by label designs. The lower the score, the more usable the participant believed the label to be. We reverse code the TLX performance metric to keep consistency with the other scales (i.e., the lower the performance score, the higher the perceived performance).

Task Load Index						
	Mental Demands	Temporal Demands	Effort	Frustration	Performance
Binary	10.57 (3)	7.36 (2)	12.32 (3)	13.00 (4)	13.02 (4)
Graded-Shield	8.92 (2)	7.52 (3)	9.66 (2)	9.29 (2)	8.69 (3)
Nutrition-Style	13.69 (4)	7.95 (4)	13.18 (4)	11.33 (3)	8.57 (2)
Five-Lock	**6.10** (1)	**6.82** (1)	**6.83** (1)	**6.44** (1)	**6.23** (1)

**Table 8:** Factors that most influence product choices. Participants ordered on a scale from 1 to 5 for Price, Privacy, Security, Brand, and Other (an open field text box capturing responses such as reliability, aesthetics, performance, and simplicity). Price was ordered 1st as a primary determinant of purchase 39.2% of the time. The averages (mean, median, mode) are the total distribution of responses. Recall that participants also demonstrated a much better understanding of label factors that impinged privacy when asked to select a correct definition.

Procurement Influence					
	Price	Privacy	Cybersecurity	Brand	Other
1	39.2%	21.1%	25.8%	10.1%	3.8%
2	16.3%	37.4%	28.2%	14.9%	3.2%
3	21.3%	27.2%	26.2%	21.5%	3.8%
4	20.1%	11.9%	16.3%	44.5%	7.2%
5	3.2%	2.4%	3.4%	8.9%	82.1%
Mean	2.32	2.37	2.43	3.27	4.61
Median	2	2	2	4	5
Mode	1	2	2	4	5

**Table 9:** Preferences of labels pre and post-study; each label design was used three times during the study to make product selections. The initial label designs are the beginning of a process, not a conclusion. A significant change in label preference is indicative of design usability.

Label Preferences				
	Pre-Study	Post-Study	% Change	Sig.
Binary	13.9%	2.4%	−11.5%	**<.001**
Graded-Shield	38.8%	16.9%	−21.9%	**<.001**
Nutrition-Style	**44.1%**	34.4%	−9.7%	**<.001**
Five-Lock	3.2%	**46.3%**	**+43.1%**	**<.001**

**Table 10:** Pre-Study response gravitated toward product design features as indicating security. Participants noted the Graded-Shield label originating from UL as a sign of security assurance; however, the majority assumed “gold” was the highest ranking for this label and therefore provided the greatest assurance. Additionally, the Nutrition-Style label having the most transparent information made it desirable in a one-shot design.

Pre-Study Responses						
	Binary	Graded-Shield	Nutrition-Style	Five Lock	Count	Percent
Transparency of Information	0	0	144	0	144	28.5%
Technical Specifications	1	2	19	0	22	4.4%
Security Factors	3	3	19	0	25	5.0%
Device Design and Aesthetics	50	34	20	1	111	22.0%
Gold or Verified	0	144	0	0	144	28.5%
Simple to Use	0	0	0	0	0	0.0%
Trustworthiness	8	3	2	8	21	4.2%
QR Codes	0	0	0	0	0	0.0%
Uncertain or Other	8	10	19	1	38	7.5%

**Table 11:** After using the labels, most participants preferred simpler, more intuitive designs. Some participants even noted that the Nutrition-Style label was “simple” because it provided specific information they could use as a point of difference.

Post-Study Responses						
	Binary	Graded-Shield	Nutrition-Style	Five Lock	Count	Percent
Transparency of Information	0	0	139	10	149	29.5%
Technical Specifications	0	0	7	1	8	1.6%
Security Factors	0	4	5	2	11	2.2%
Label Design and Aesthetics	6	7	4	0	17	3.4%
Gold or Verified	0	19	0	0	19	3.8%
Simple to Use	0	44	12	200	256	50.7%
Trustworthiness	3	8	3	1	15	3.0%
QR Codes	0	0	0	15	15	3.0%
Uncertain or Other	3	4	4	4	15	3.0%

**Figure 17:**
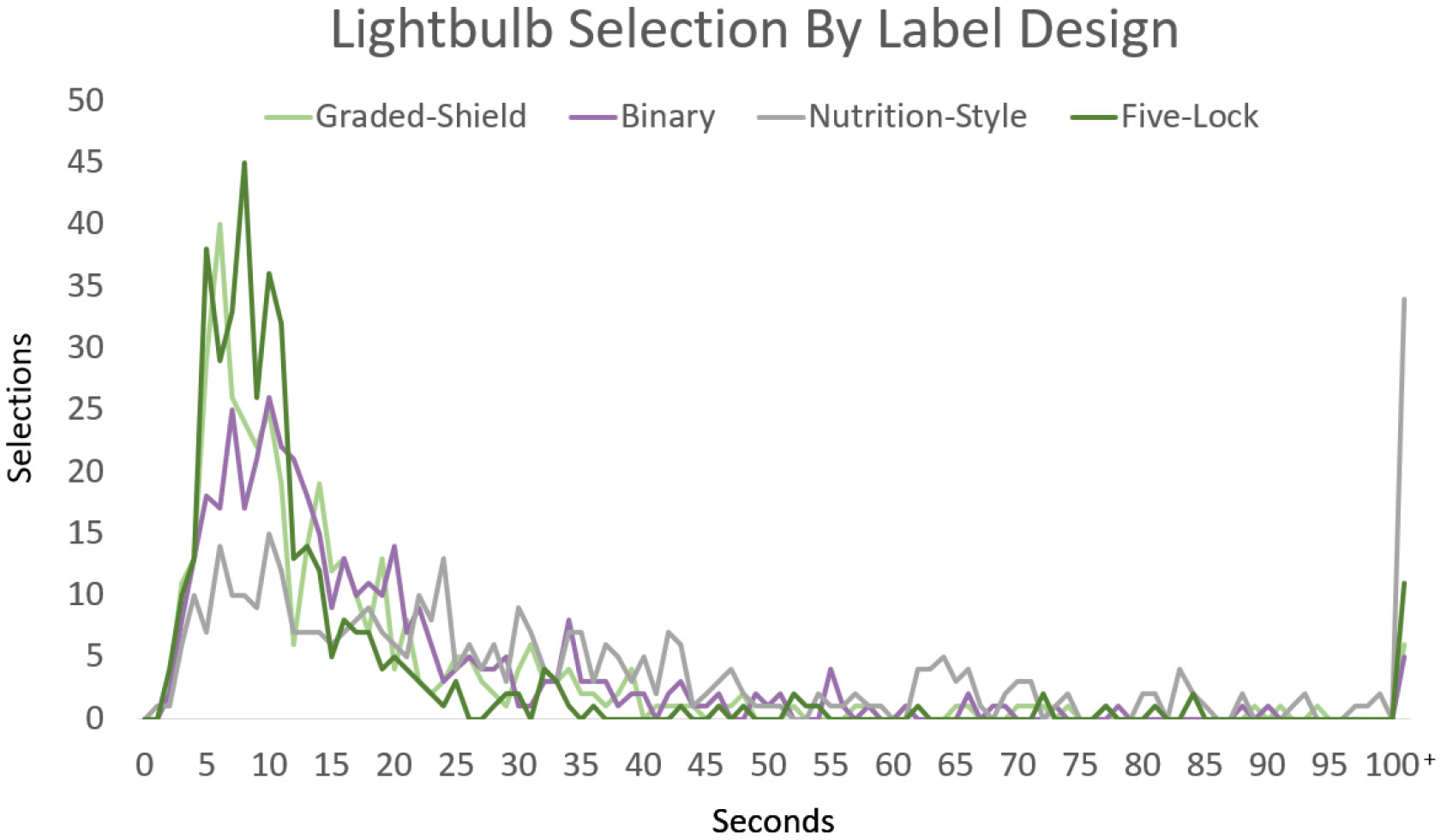
Time to selection across all four label designs. After 100 seconds, responses are grouped. We can see that the Nutrition-Style label required the most time to select, with 34 participants taking longer than 100 seconds.

**Figure 18:**
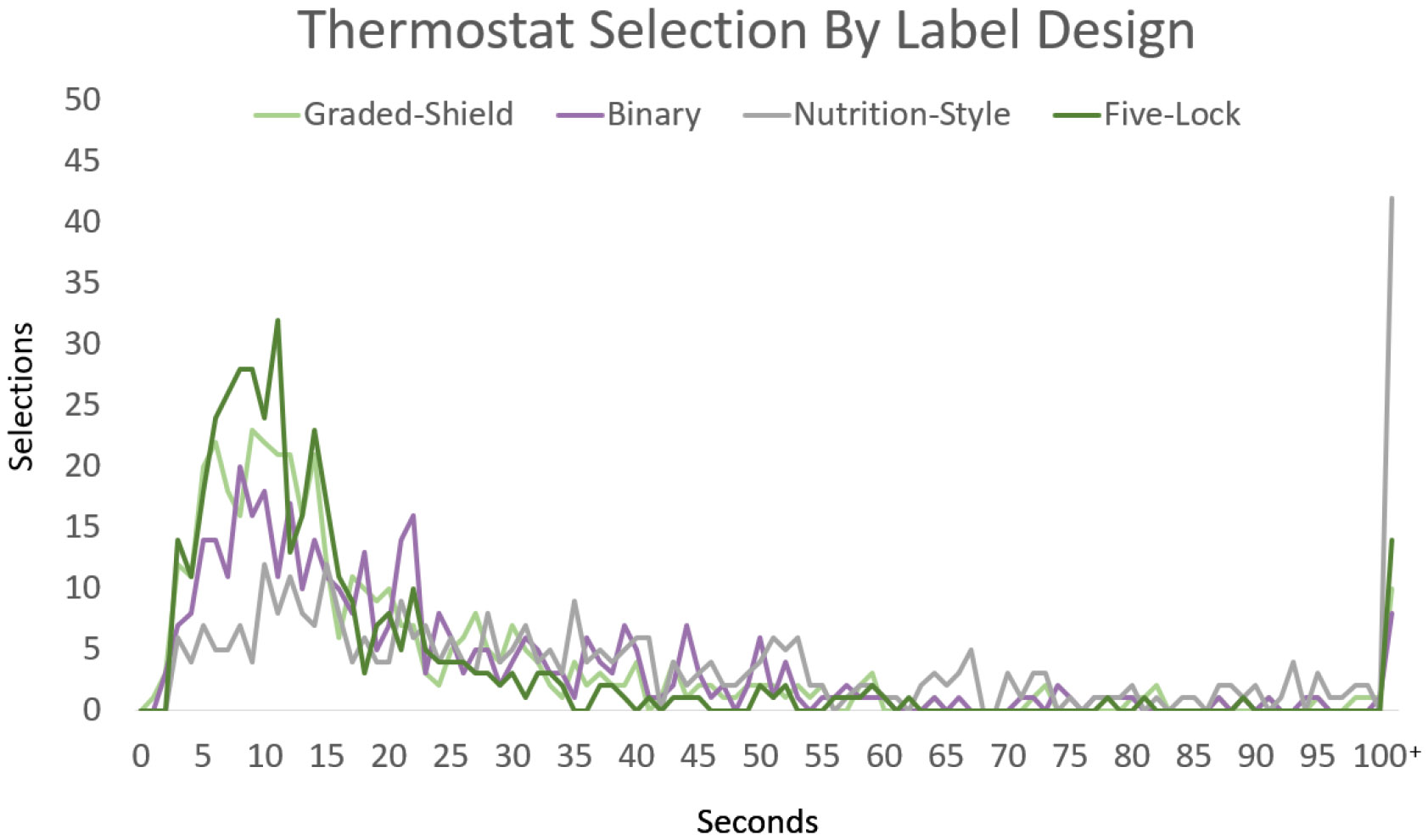
Time to selection across all four label designs. After 100 seconds, responses are grouped. We can see that the Nutrition-Style label required the most time to select, with 42 participants taking longer than 100 seconds.

**Figure 19:**
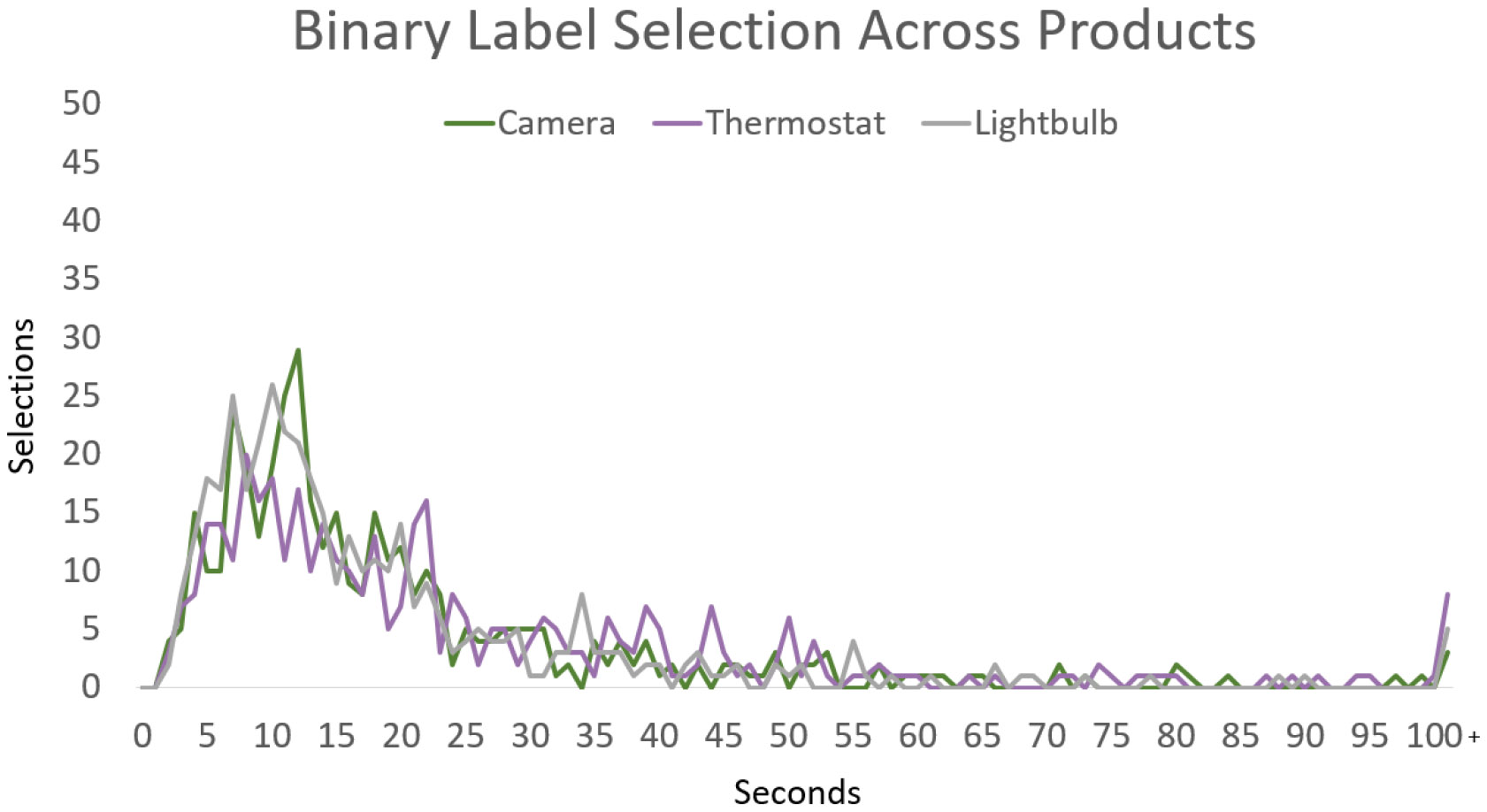
Binary labels across Cameras, Thermostats, and Light Bulbs had a mean of 22.3 seconds, a median of 14.6 seconds, and a standard deviation of 54.77 seconds.

**Figure 20:**
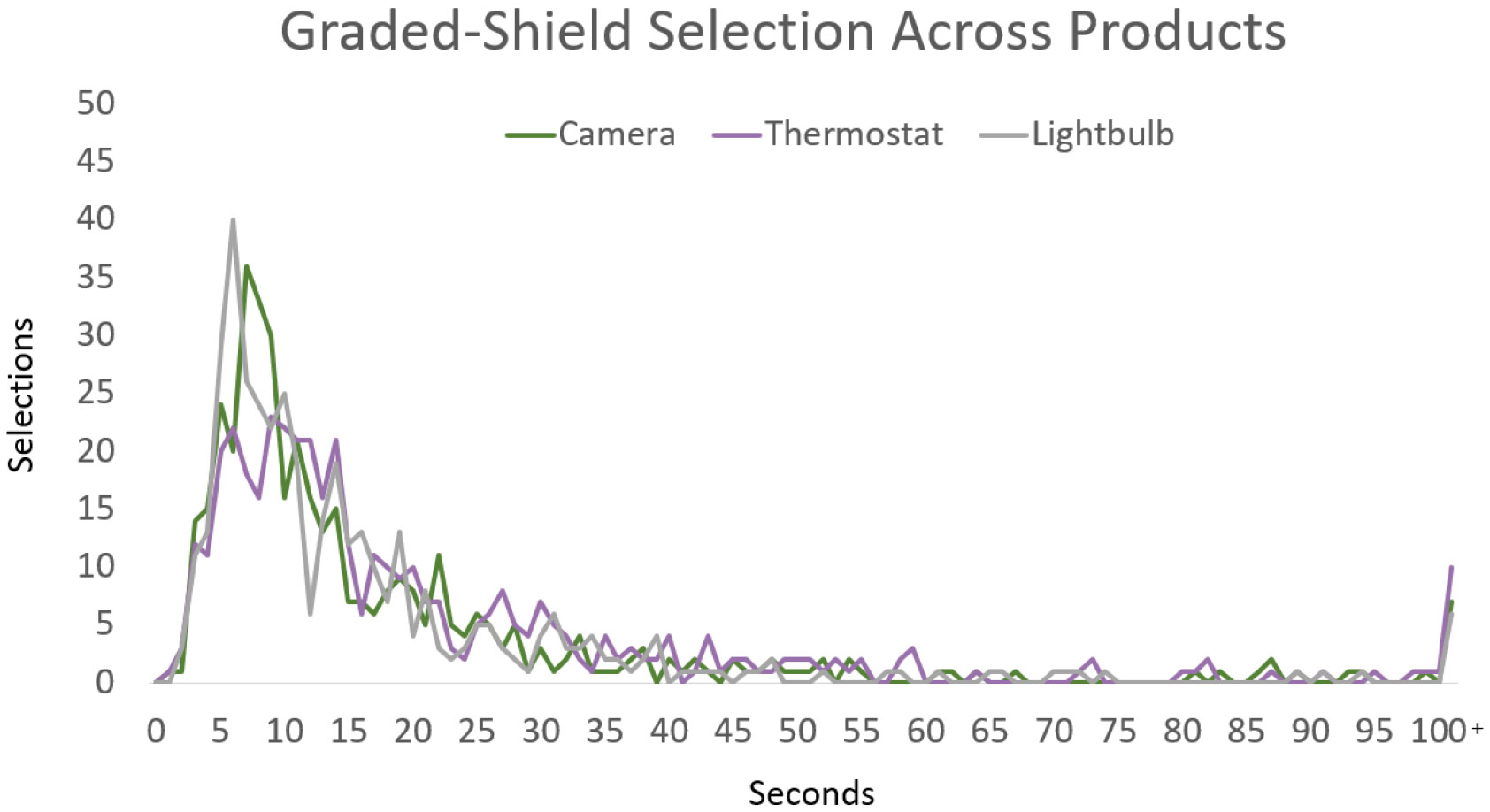
Graded-Shield labels across Cameras, Thermostats, and Light Bulbs had a mean of 19.8 seconds, a median of 12.0 seconds, and a standard deviation of 25.31 seconds.

**Figure 21:**
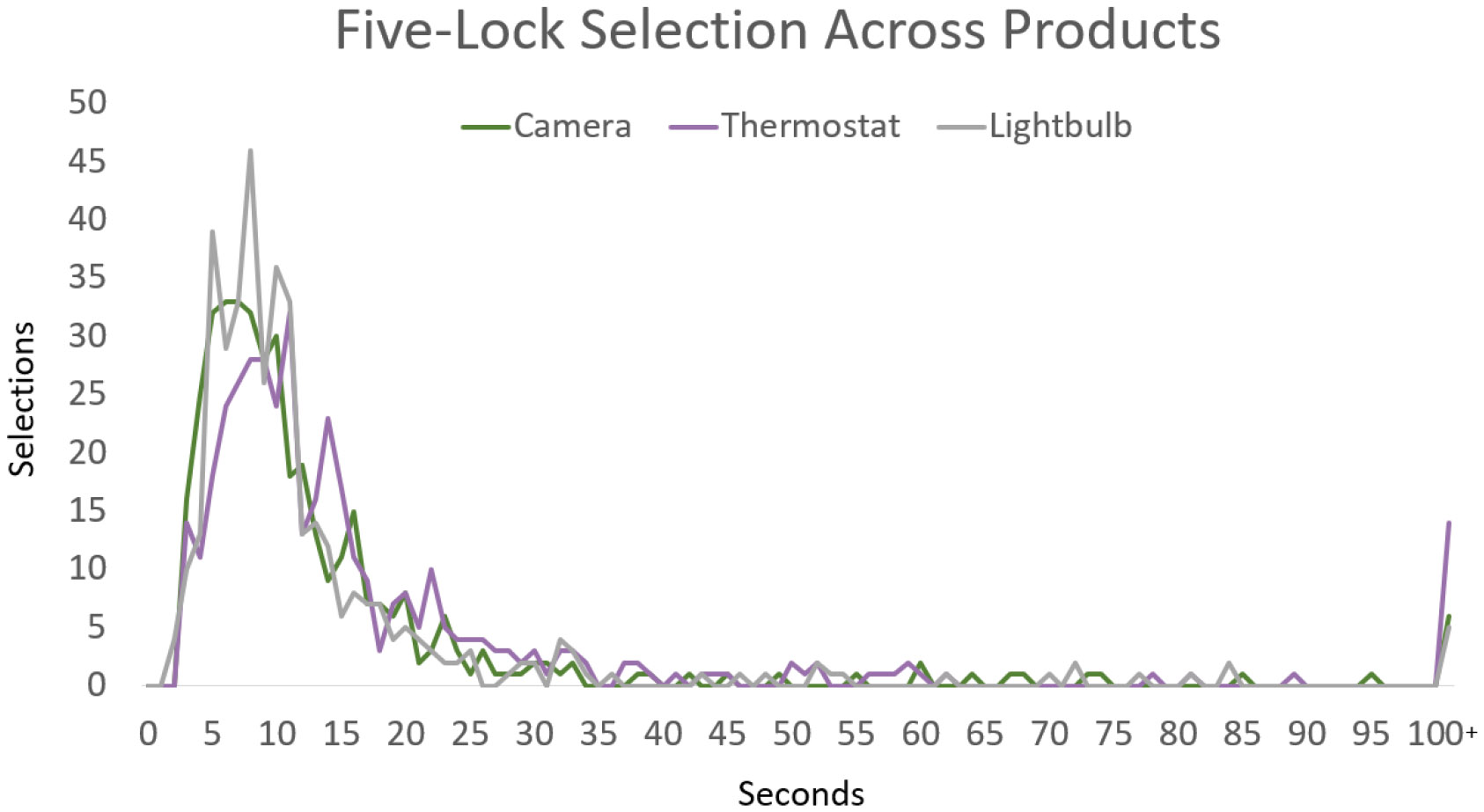
Five-Lock labels across Cameras, Thermostats, and Light Bulbs had a mean of 19.03 seconds, a median of 9.9 seconds, and a standard deviation of 56.28 seconds.

**Figure 22:**
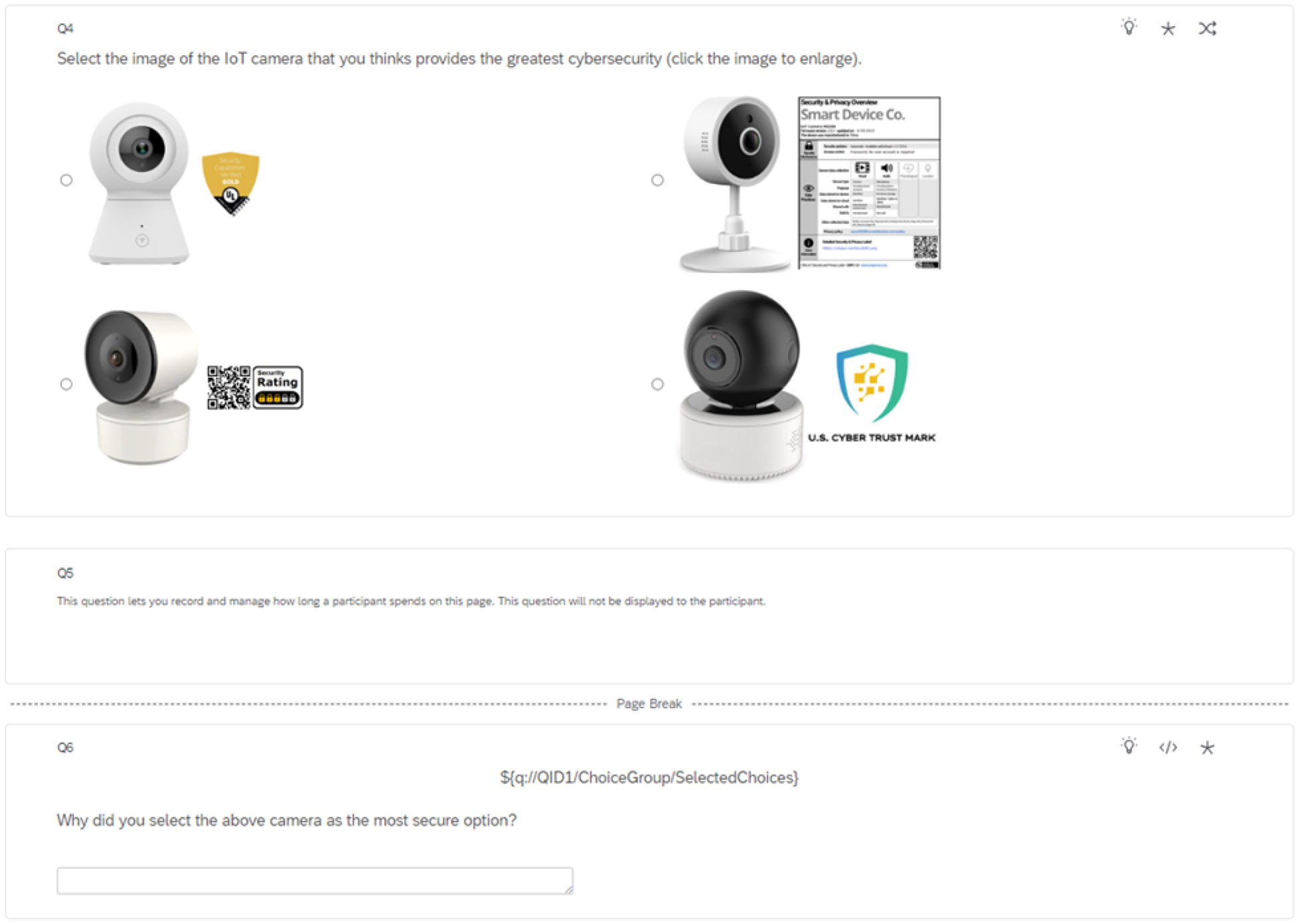
Phase 2 of the study is designed to gauge initial preference for security label designs. Label designs represent medium security and are randomly assigned to the camera devices.

**Figure 23:**
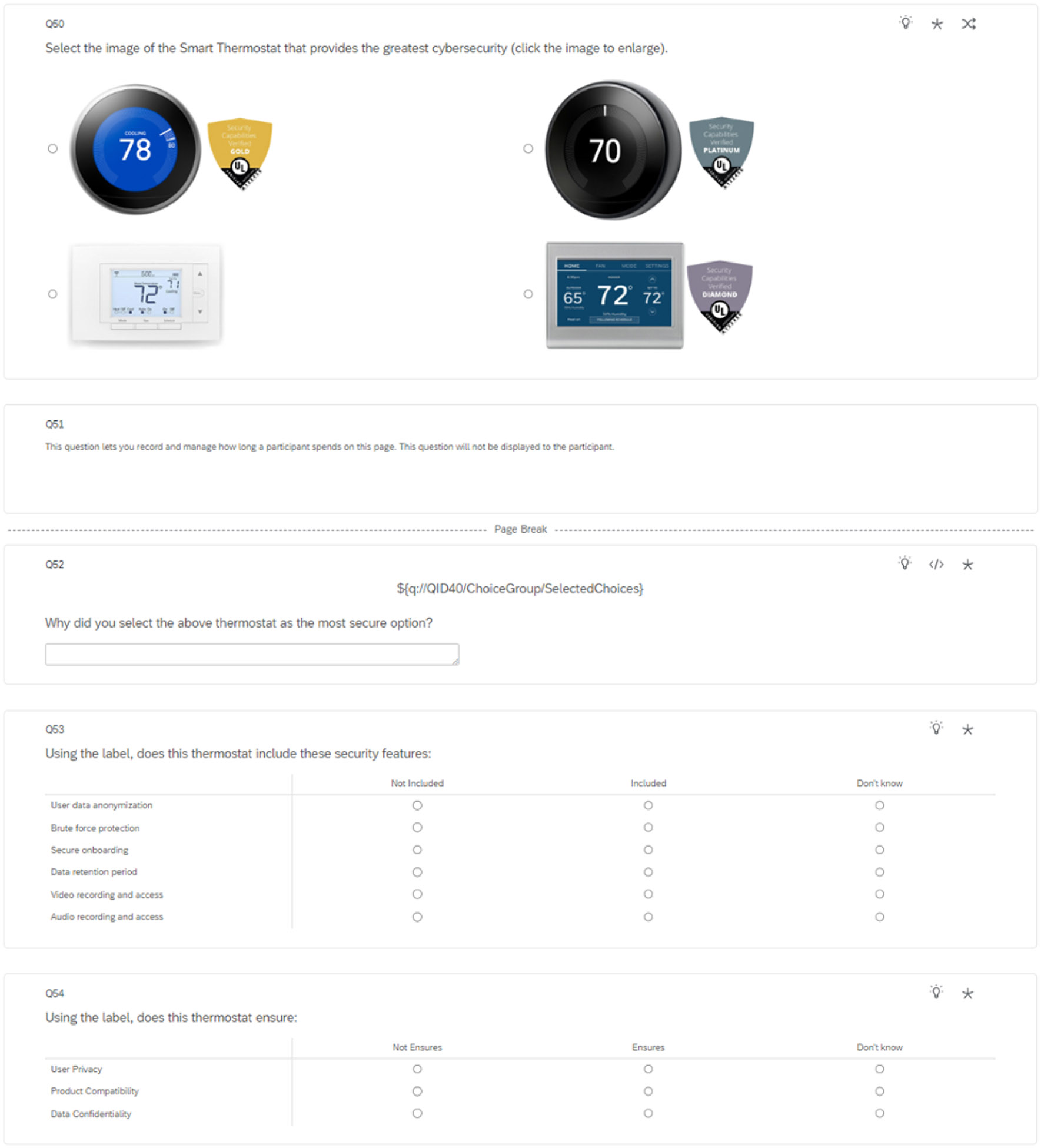

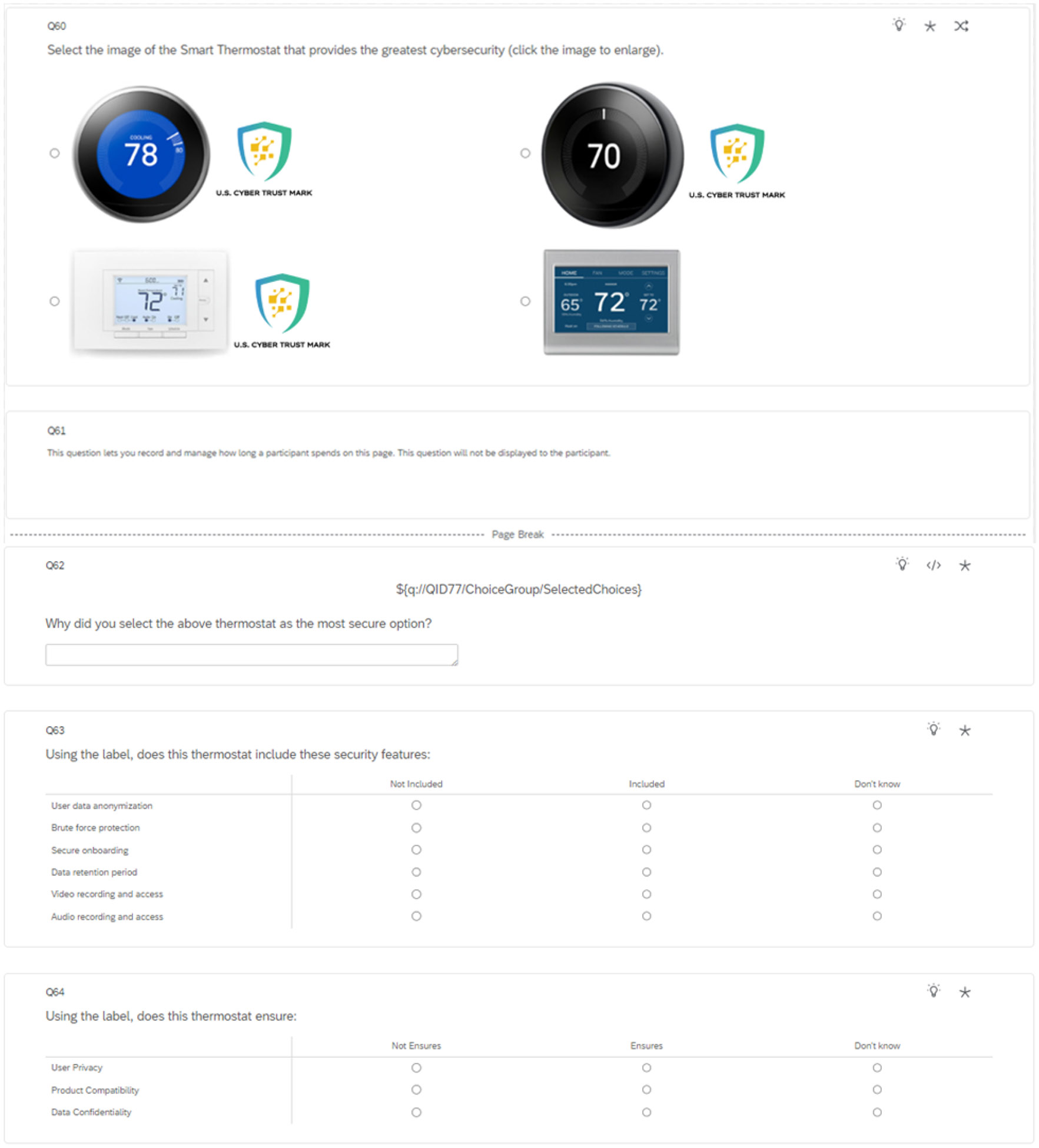

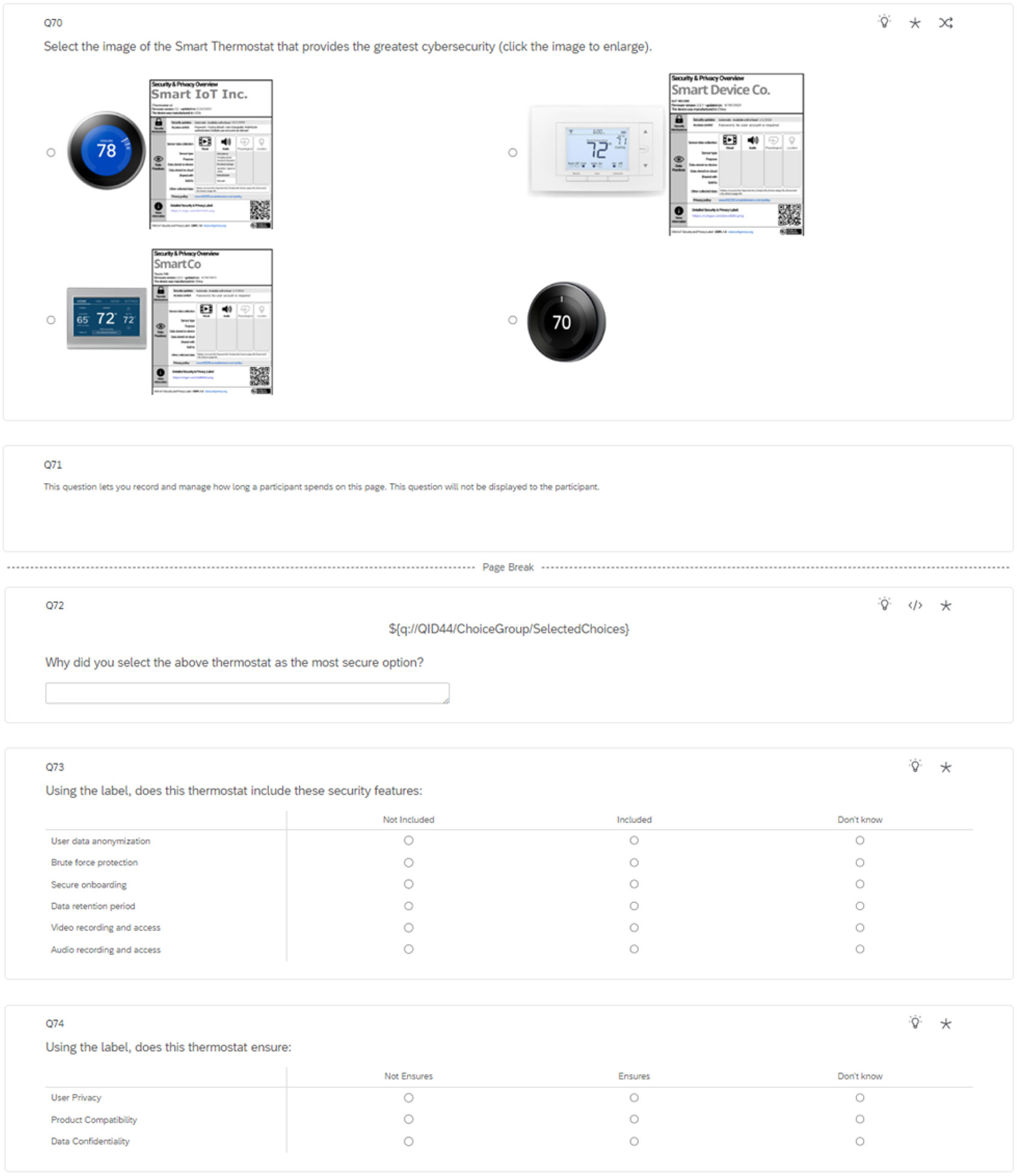

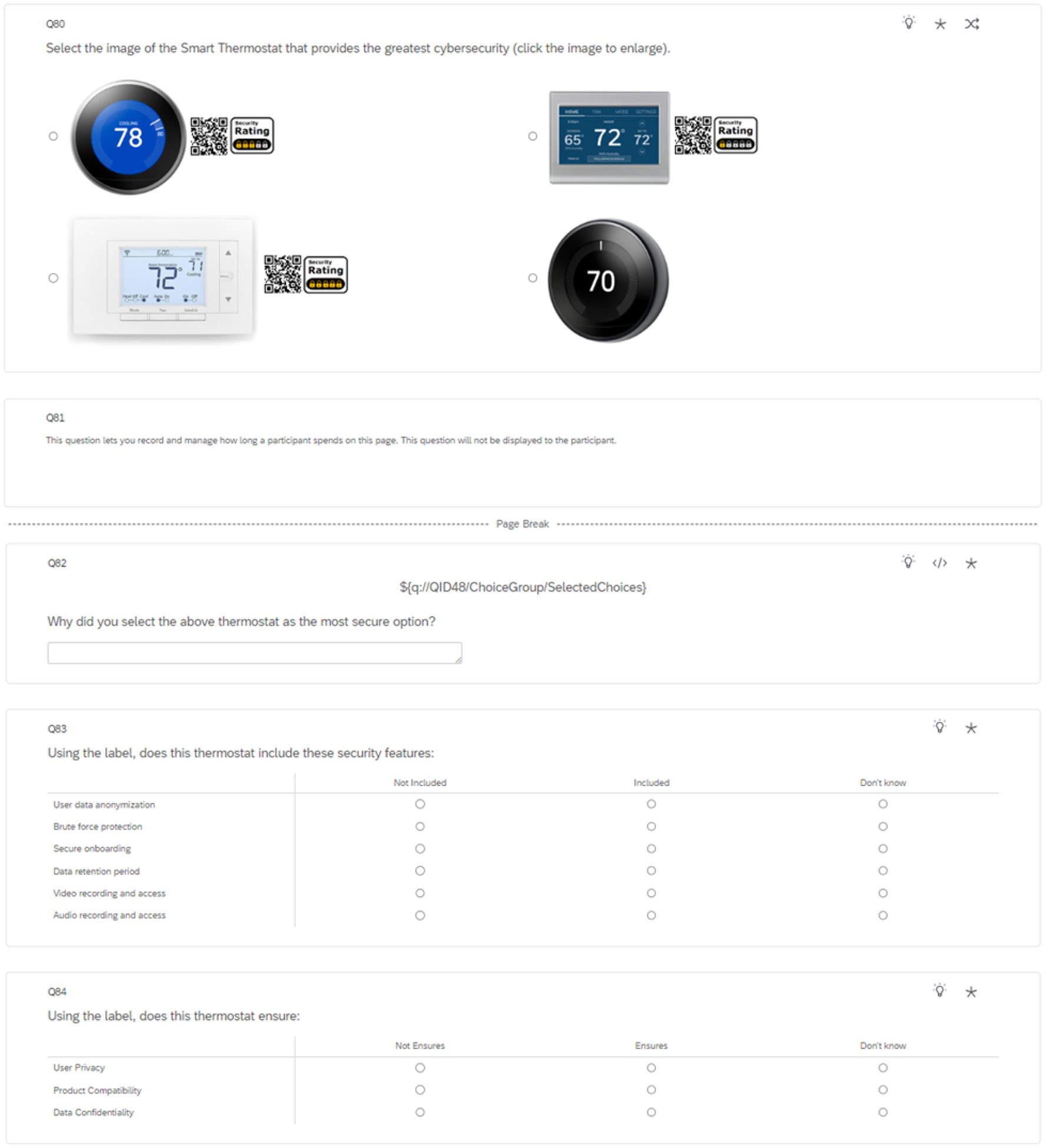
Phase 3 of the study randomly presents participants with a product type (i.e., thermostats) and a label design (i.e., Graded-Shield label). Participants are asked to choose which device appears to provide the greatest level of security, justify their response, and evaluate the inherent security qualities of the device.

**Figure 24:**
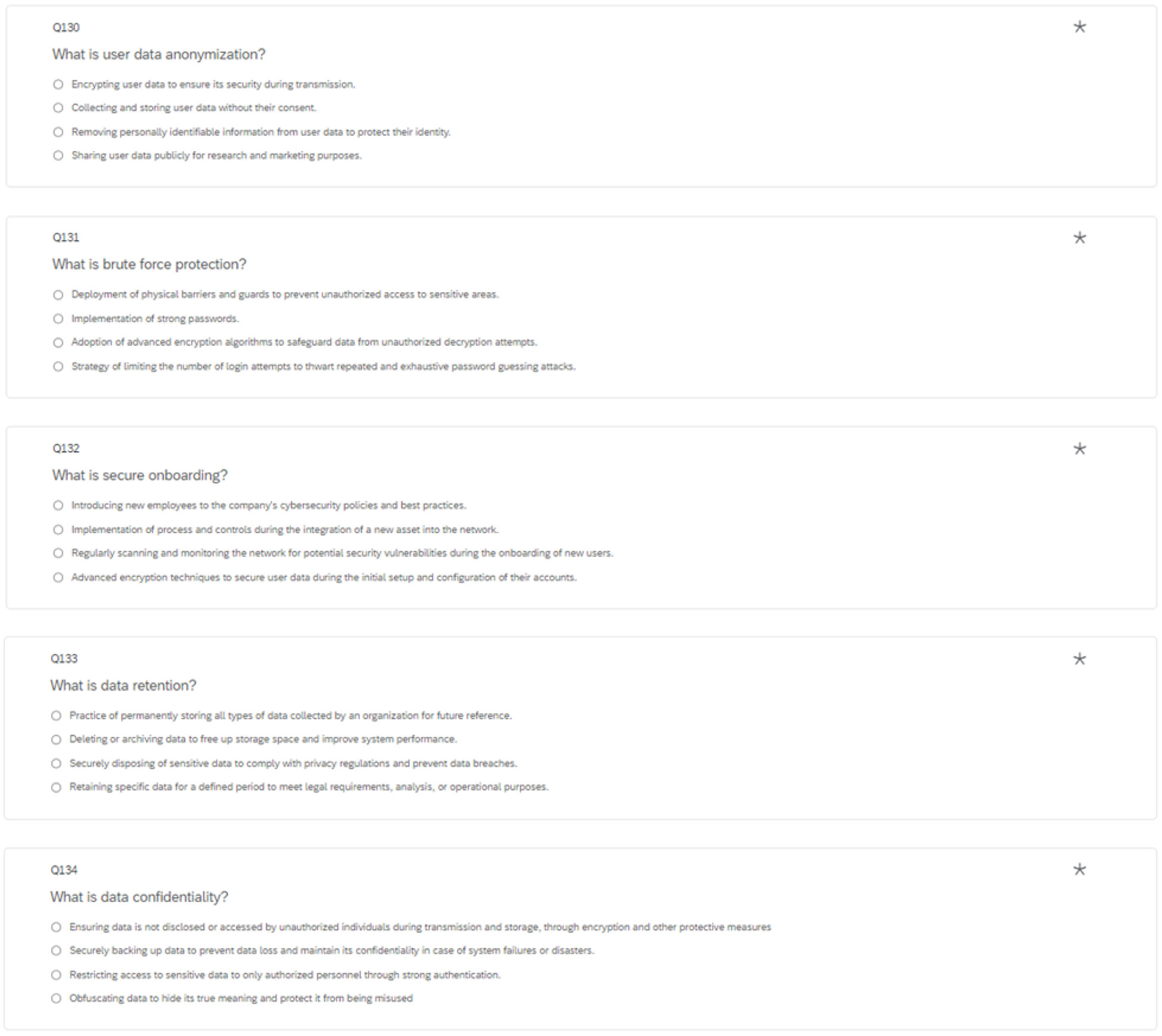
Phase 4 captures participant knowledge within the security domain.

**Figure 25:**
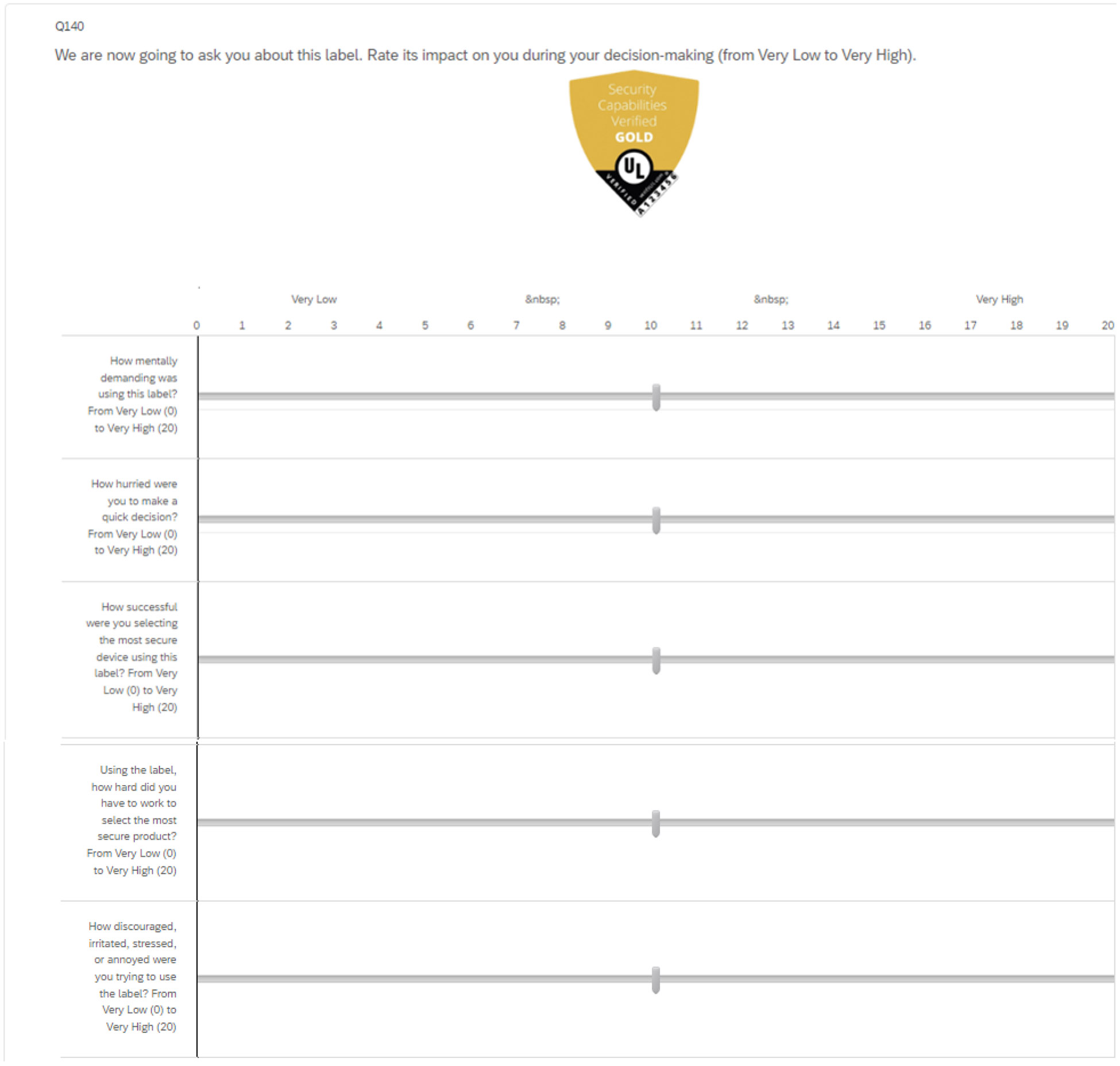
Phase 5 of the study uses NASA-TLX to measure the cognitive demands of each label design across five dimensions: mental demand, temporal demand, performance, effort, and frustration.

**Figure 26:**
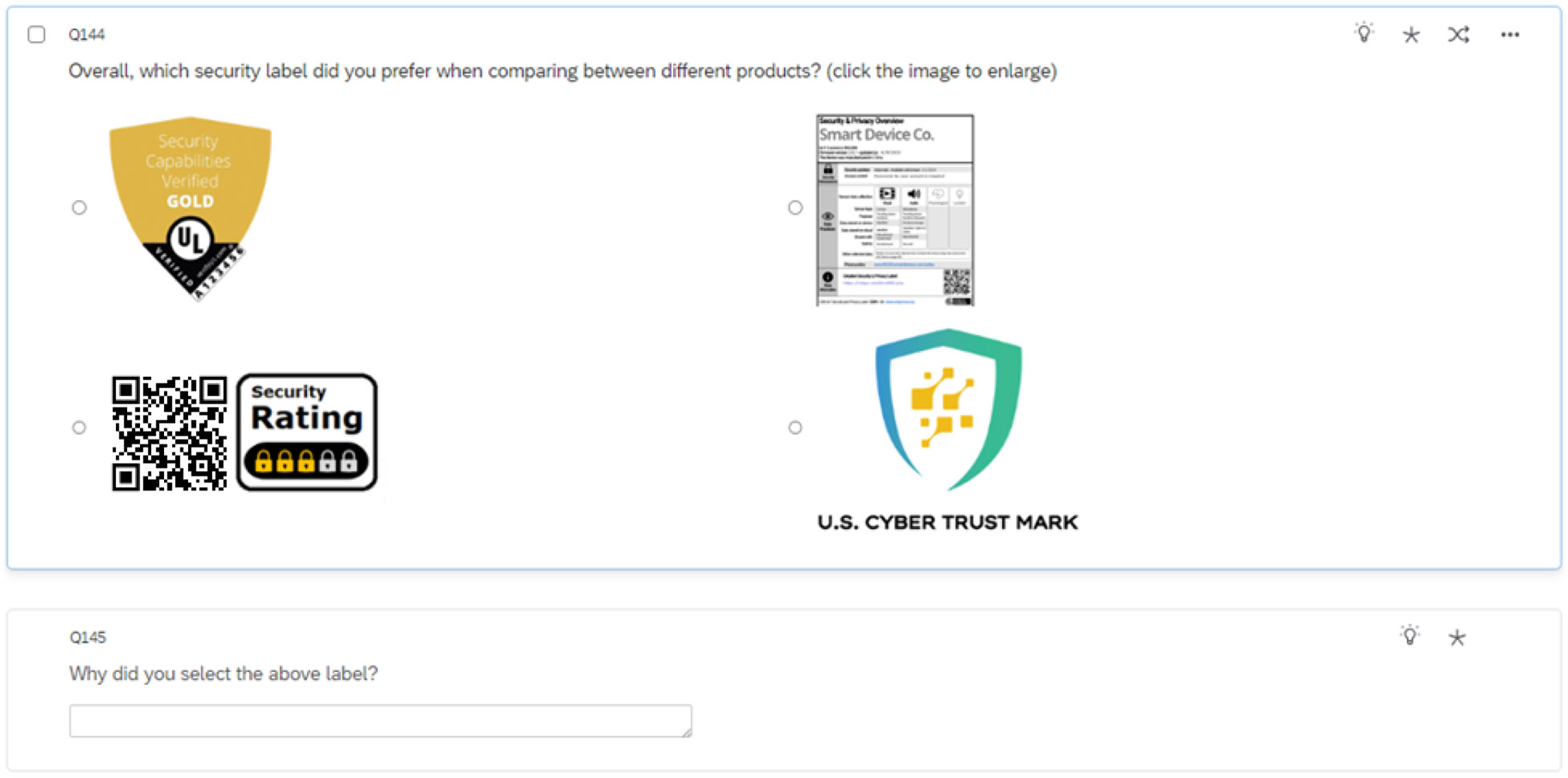
Phase 6 re-evaluates participant perception and preferences of label design after use in the study. We also capture responses to compare pre and post-study changes.

**Figure 27:**
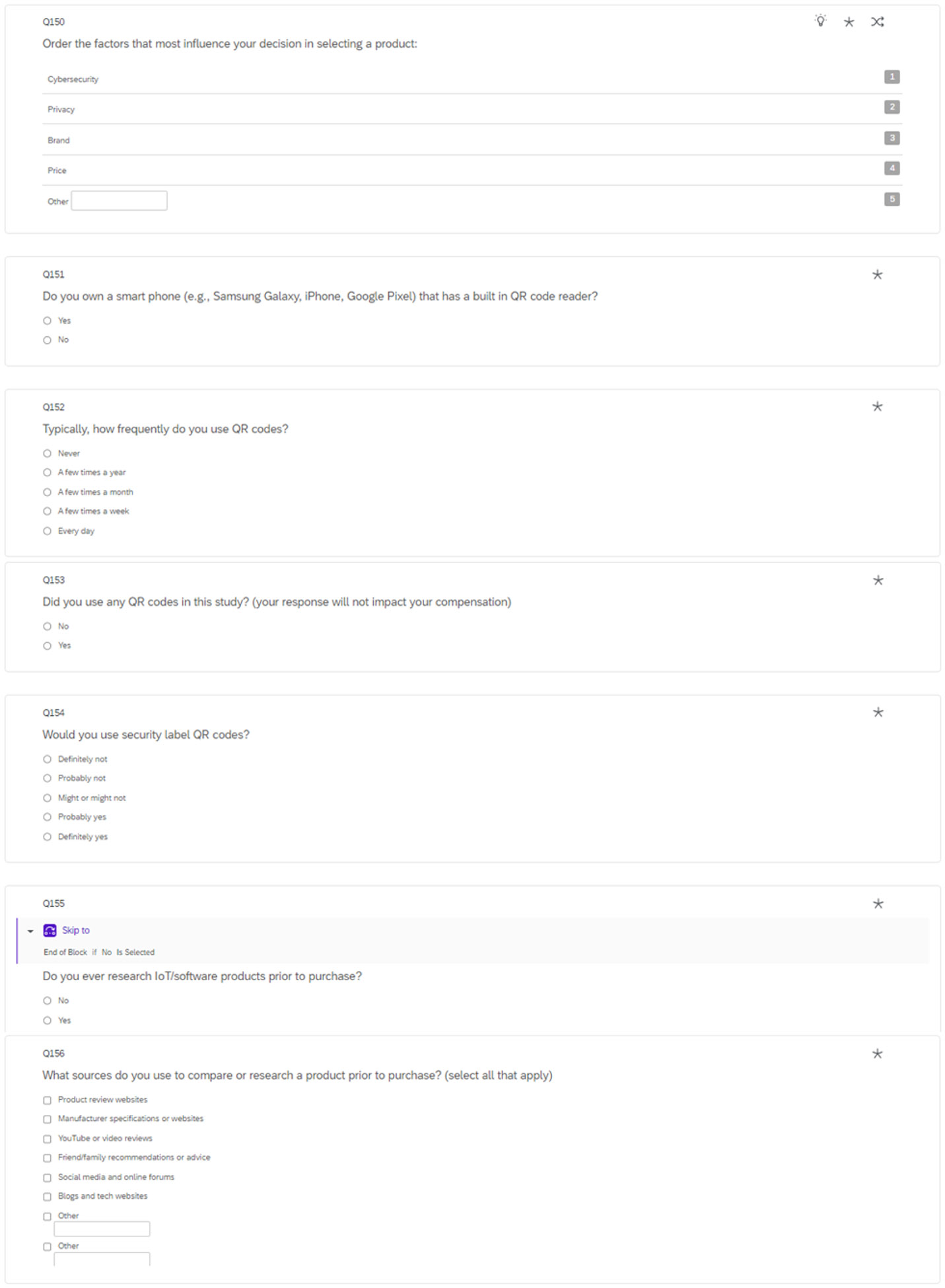
Phase 7 captures participant prefen of security and QR codes during procurement making.

**Figure 28:**
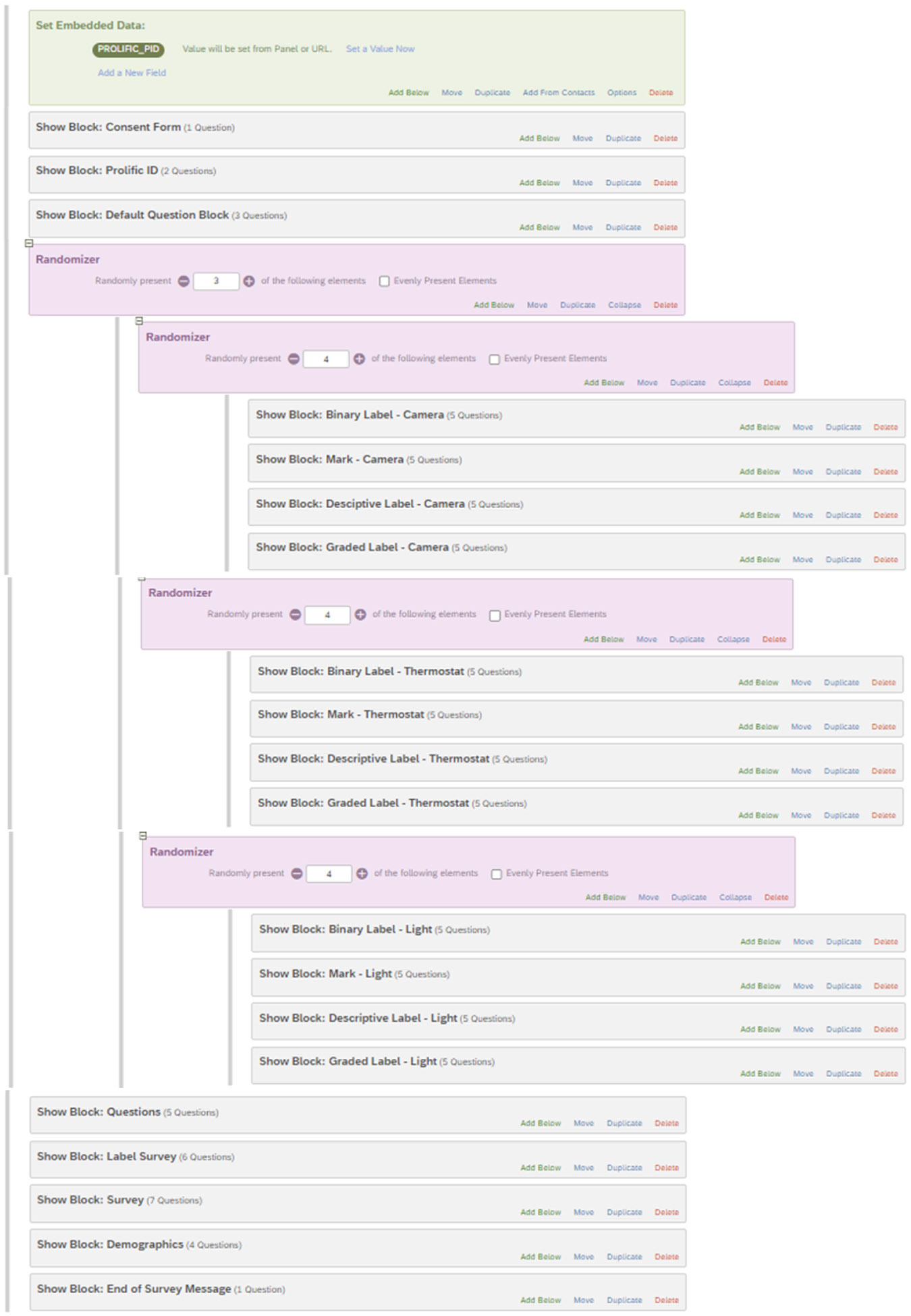
This is another representation of the survey flow used throughout the study.

## CCS CONCEPTS

• Human-centered computing → Empirical studies in HCI; • Security and privacy → Usability in security and privacy.
